# Bone marrow mesenchymal stem cells in premature ovarian failure: Mechanisms and prospects

**DOI:** 10.3389/fimmu.2022.997808

**Published:** 2022-10-27

**Authors:** Yanjing Huang, Mengdi Zhu, Zhuo Liu, Runan Hu, Fan Li, Yufan Song, Yuli Geng, Wenwen Ma, Kunkun Song, Mingmin Zhang

**Affiliations:** ^1^ Institute of Integrated Traditional Chinese and Western Medicine, Tongji Hospital, Tongji Medical College, Huazhong University of Science and Technology, Wuhan, Hubei, China; ^2^ Department of Integrated Traditional Chinese and Western Medicine, Tongji Hospital, Tongji Medical College, Huazhong University of Science and Technology, Wuhan, Hubei, China

**Keywords:** premature ovarian failure, mesenchymal stem cells, ovary, ovarian function, bone marrow

## Abstract

Premature ovarian failure (POF) is a common female reproductive disorder and characterized by menopause, increased gonadotropin levels and estrogen deficiency before the age of 40 years old. The etiologies and pathogenesis of POF are not fully clear. At present, hormone replacement therapy (HRT) is the main treatment options for POF. It helps to ameliorate perimenopausal symptoms and related health risks, but can’t restore ovarian function and fertility fundamentally. With the development of regenerative medicine, bone marrow mesenchymal stem cells (BMSCs) have shown great potential for the recovery of ovarian function and fertility based on the advantages of abundant sources, high capacity for self-renewal and differentiation, low immunogenicity and less ethical considerations. This systematic review aims to summarize the possible therapeutic mechanisms of BMSCs for POF. A detailed search strategy of preclinical studies and clinical trials on BMSCs and POF was performed on PubMed, MEDLINE, Web of Science and Embase database. A total of 21 studies were included in this review. Although the standardization of BMSCs need more explorations, there is no doubt that BMSCs transplantation may represent a prospective therapy for POF. It is hope to provide a theoretical basis for further research and treatment for POF.

## Introduction

Premature ovarian failure (POF) refers to the decline of the ovarian function that occurs before the age of 40 in female. It is a clinical syndrome defined by oligomenorrhea or amenorrhea, increased gonadotropin levels, and decreased estradiol levels, and it is often accompanied by a variety of perimenopausal symptoms such as hot flashes, night sweats, alopecia, dry skin and mucous membranes, decreased libido, sleep disorders, irritability (1, 2). The diagnosis of POF in clinical is usually based on FSH >40 IU/L, oligo/amenorrhea for 4-6 months in female under 40 years old, and hypoestrogenemia (3, 4). In addition, the reduction of anti-Mullerian hormone is also an important auxiliary diagnostic criterion.

Women with POF have an increased risk of psychological disorders, cardiovascular diseases, osteoporosis, autoimmune diseases, cognitive dysfunction, urinary and reproductive system infections and other diseases compared with normal people. In addition, low fertility and even infertility are also major problems for POF patients (5, 6). Statistics showed that the incidence of POF was about 1% in female before the age of 40. The incidence of POF is on the rise due to the younger age of cancer onset, environmental pollution, lifestyle changes and other factors. Nevertheless, the etiology of POF is complex and not fully understood. Current studies show that the pathogenic factors of POF include iatrogenic factors (chemotherapy, radiotherapy, pelvic surgery, etc.), X chromosome abnormality, genetic syndrome, single gene mutation, congenital enzyme deficiency, autoimmune diseases, infection, HPV vaccination, environmental influence, etc. (5, 7). Complex clinical symptoms and adverse consequences caused by POF have greatly affected the quality of life of patients. Exploring effective treatment of POF has been a goal of clinical and scientific researchers.

Currently, there is no effective treatment for POF. HRT is the main therapeutic schemes for POF, which can effectively improve the menopause symptoms and reduce the risk of osteoporosis and cardiovascular diseases, as well as improve the quality of life of patients. However, HRT can’t fully restore ovarian function, such as hormone secretion, follicular growth or ovulation (8). Moreover, it is not entirely clear that whether or not HRT increases the risk of breast cancer (9). Ovarian tissue cryopreservation is a novel treatment for POF. Nonetheless, there are many problems with ovarian tissue after cryopreservation such as low survival rate and difficulty in natural conception (10). The common treatments for POF include psychological support, melatonin, androgen or dehydroepiandrosterone supplementation, traditional Chinese medicine therapy, diet and exercise conditioning, immune regulation, etc., but none of them can fundamentally improve ovarian function and meet the fertility needs of patients (7, 11). To protect POF patients from the disease, researchers have been exploring new treatments in recent years, such as perfusing platelet-rich plasma into the ovaries, ovarian tissue transplantation, building artificial ovary, artificial gametes and mitochondria replacement therapy, etc., which provide a new way for treating POF. However, they are limited by high cost, poor practical application and ethics (12).

Stem cell therapy has made great strides in regenerative medicine over the past two decades. “Stem cells” refer to the specific cell types that are insufficiently differentiated and immature, capable of self-renewal, which can proliferate and differentiate into various tissues and organs. Based on the therapeutic potential of stem cells in multiple systems, exploring the potential role of stem cells in treating female reproductive system diseases has become the focus of cutting-edge research. Recent years, a number of studies have also confirmed that many stem cells are effective in treating POF, including mesenchymal stem cells (MSCs), ovarian germ stem cells (OGSCs), embryonic stem cells (ESCs). Among them, BMSCs have shown great potential in repairing ovarian damage and restoring ovarian function in POF animal models or patients (13, 14). Due to their advantage of self-renewal capacity, multipotency, low immunogenicity, injury chemotaxis and less ethical controversy (15–18). BMSCs show a great therapeutic prospect in POF. Therefore, the mechanism and research progress of BMSCs in the treatment of POF are reviewed below.

## Methods

The study was carried out following the PRISMA guidelines (19). Keywords and their combinations included: (Mesenchymal Stem Cell) OR (Stem Cell, Mesenchymal) OR (Bone Marrow Mesenchymal Stem Cell) OR (Bone Marrow Stromal Cell) OR (Mesenchymal Stromal Cell) OR (Stromal Cell, Mesenchymal) OR (Multipotent Mesenchymal Stromal Cell) OR (Mesenchymal Progenitor Cell) AND (Primary Ovarian Insufficiency) OR (Premature Ovarian Failure) OR (menopause, premature). The search strategy was applied to PubMed, MEDLINE, Web of Science and Embase database. The filters included: full text and female and the publications in the English language and year=“2005-Current”. The abstracts of the articles were included as following criteria (1): BMSCs transplantation in treating premature ovarian failure (2). Only original research articles were included, but not reviews.

**Figure 1:**
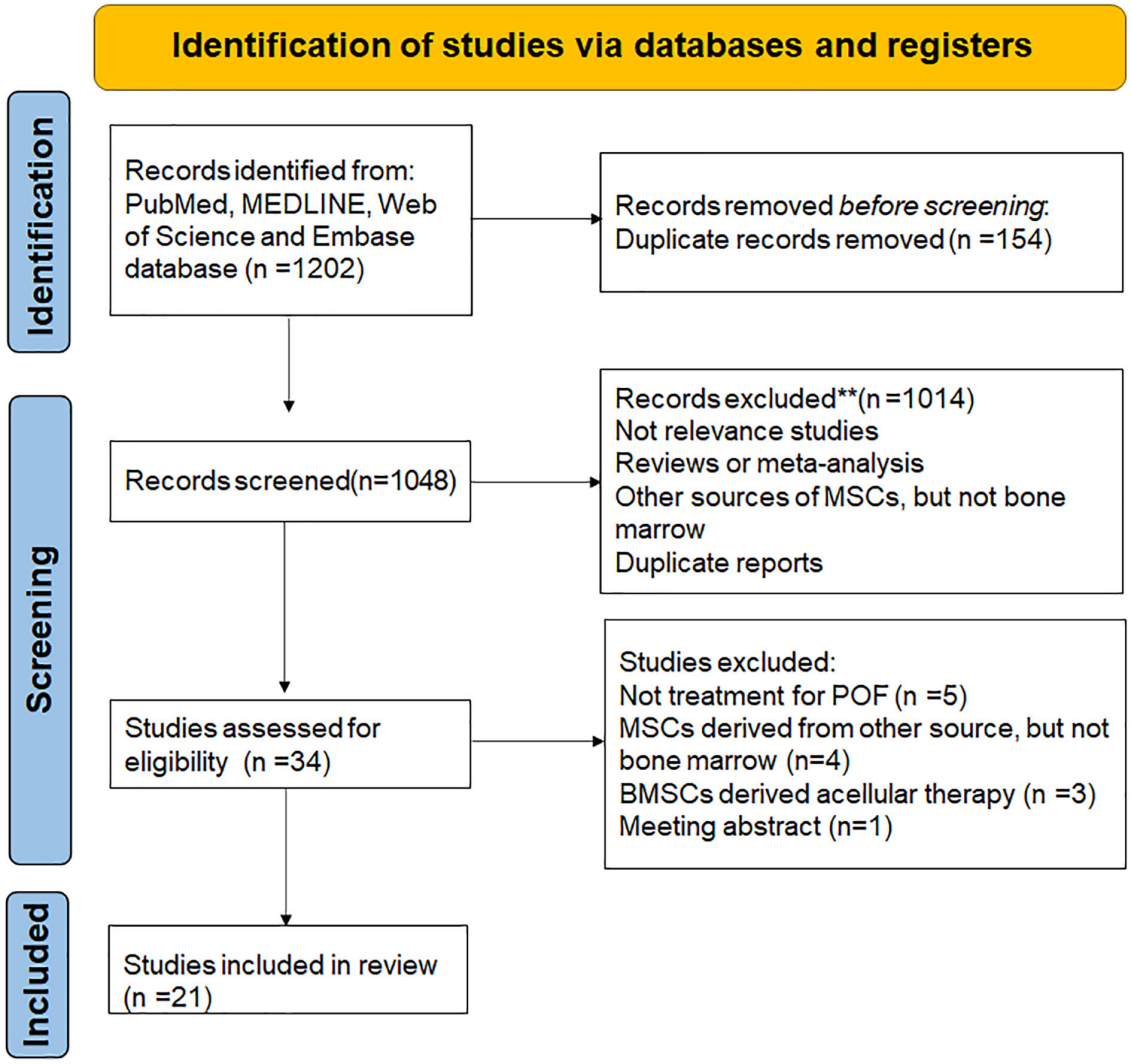
PRISMA flow diagram.

A total of 1202 articles were retrieved after the initial search. 154 duplicate records were removed after importing into endnote software. After screening the title and abstract, 1014 articles were excluded mainly because they were not relevance with the current analysis, or they were reviews or meta-analysis, or other sources of MSCs but not bone marrow, or duplicate reports. Among the 34 potentially relevant studies, 14 were further excluded after reviewing full texts due to 5 studies were unrelated to the treatment of POF, 4 studies were unrelated bone marrow derived MSCs, 3 studies were related to bone marrow derived acellular therapy and one paper was meeting abstract (**Figure 1**).

## Biological characteristics of BMSCs

MSCs are a kind of pluripotent stem cells originating from the early mesoderm, which can be isolated from bone marrow, adipose, dental pulp, placenta, umbilical cord, amniotic membrane, amniotic fluid and other tissues (20). Among them, bone marrow is the most important source of MSCs (21).

In the mid-1960s, Friedenstein et al. first identified BMSCs in mouse bone marrow, which were characterized by fibroblast-like cells with clonal potential (22). In recent decades, BMSCs have attracted plenty of attention for their potential in regenerative medicine and tissue engineering in replacing, repairing or restoring the function of damaged tissues or organs. Among the 1102 studies on “bone marrow stem cell” in the U.S. National Library of Medicine database, there are 95 studies of Phase III and 20 studies of Phase IV showing higher potency of BMSCs in clinical application than other stem cell types (23). Although MSCs are widely used in regenerative medicine, tissue engineering and immune regulation, defining for MSCs basing on surface markers and differentiation potential has so far been fragmentary (24). Depending on the minimum criteria of define MSCs published by the International Society for Cellular Therapy and recent studies, MSCs are defined as follows: first, the growth of cells *in vitro* will adhere to the substrate; second, the cells are characterized by expressing CD105, CD90, CD73, CD44 and Sca1 surface antigens, while lack of CD34, CD45, CD14 or CD19, CD79α, CD11b and HLA-DR; Meanwhile, these cells must have the ability of differentiating towards osteoblasts, chondroblasts and adipocytes *in vitro (* 25–27). In addition to the surface markers mentioned above, the following antigens, including CD9, CD10, CD13, CD29, CD49, CD51, CD54, CD117, CD146, CD166 and Stro-1 are also expressed on the surface of MSCs (28). However, the specific combination expression of these markers varies with different host tissues (28). As mentioned above, a variety of positive markers of MSCs have been identified, but the specific markers of MSCs have not been found yet. Even though the cells meet the minimum standards of defining MSCs, there are great differences in their transcription patterns and differentiation potential *in vitro (* 29). For example, human BMSCs express CD29, CD44, CD73, CD90, CD105 and Sca1, while lack of expression CD14, CD34, CD45, CD19, CD11b, CD31, CD86, Ia and HLA-DR, but the human adipose-derived MSCs (ADMSCs) are not completely identical with BMSCs, which express CD29, CD44, CD73, CD90, CD105, CD146, CD166 and MHC-I but not CD31, CD45 and HLA-DR (30). Single-cell experiments showed that there are differences in metabolic pattern, stress response and immunogenicity between BMSCs and adipose MSCs, and moreover, BMSCs were more heterogeneous (29). Andrzejewska and Chu et al. also found that the characteristics of BMSCs are tightly related to the age or pathological status of the donor (31). With the increase of age, the number of BMSCs and their potential of adipogenic, chondrogenic and osteogenic differentiation will decrease, meanwhile, the marker phenotype or stress level markers will also change (23, 31). Hass R et al. confirmed that compared with adult-MSCs, neonatal tissue-derived MSCs may have notable biological properties, such as higher multiplication capacity, differentiation potential and life span (32). In addition, depending on the source, the immunophenotypic and secretome vary from different MSCs that accounts for some differences in their responses (33, 34). BMSCs have been widely used due to their great potential of proliferation and multidirectional differentiation as well as stable genetic background (35–38). Recent studies also confirmed that BMSCs have shown remarkable therapeutic effects in many diseases including hematopoietic diseases, musculoskeletal diseases, immune disorders, neurodegenerative diseases, cardiovascular diseases, sports injuries, gastrointestinal and cutaneous diseases as well as POF (39–44). The therapeutic mechanisms of BMSCs are diverse. First, they can secrete a variety of soluble factors, including cytokines, growth factors and chemokines as well as immunomodulatory molecules, which participate in regulating proliferation, apoptosis, fibrosis and immune regulation of damaged tissues. In addition, BMSCs can also home to the damaged tissue and differentiate into specific cells to reconstruct the damaged local microenvironment, so as to maintain the integrity of tissue morphology and function stability (35, 45–47).

## The mechanism of BMSCs in treating POF

**Figure 2:**
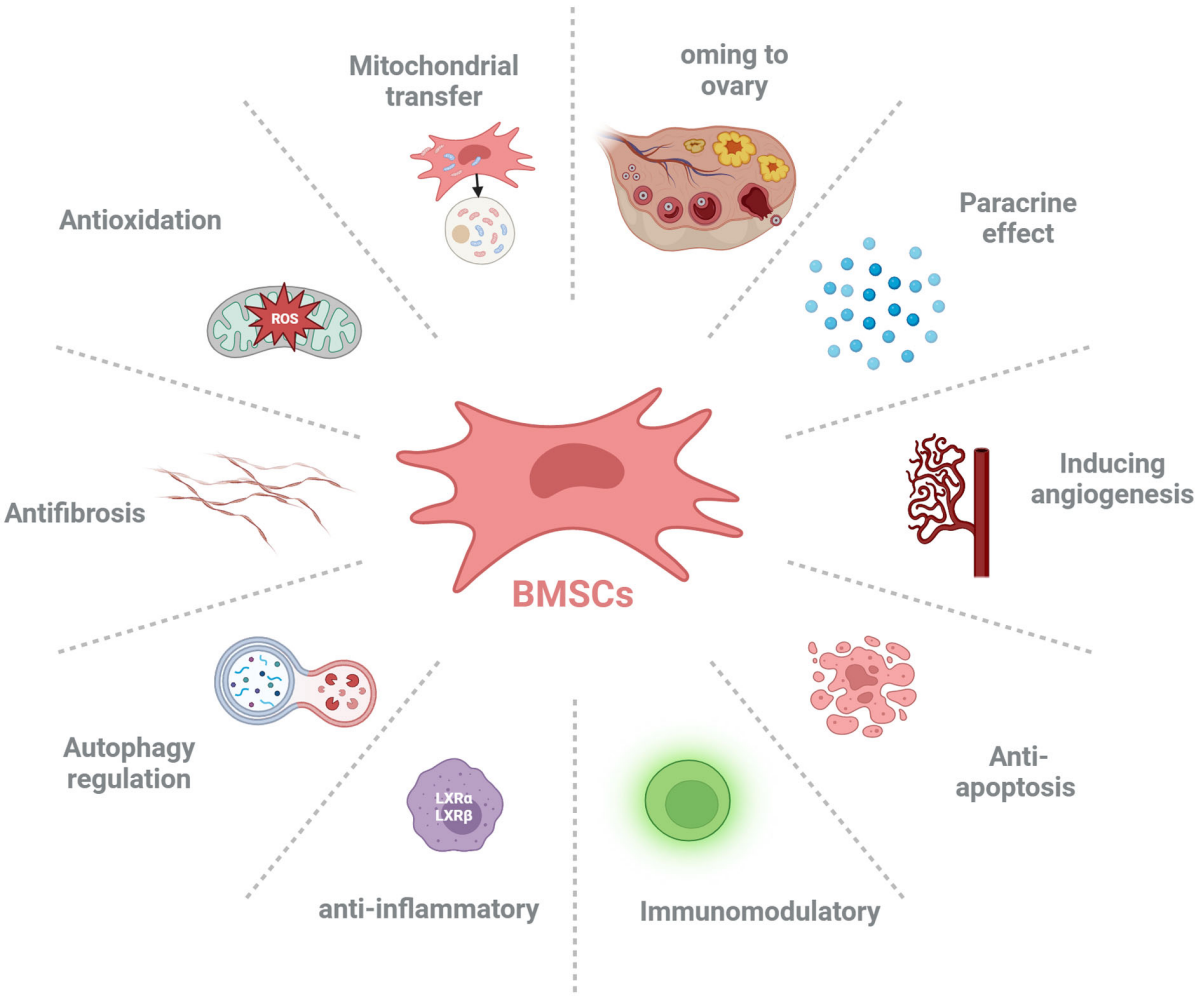
The possible mechanisms of bone marrow-derived mesenchymal stem cells (BMSCs) ameliorate premature ovarian failure (POF). BMSCs ameliorate ovarian function of POF through homing to injured ovary, paracrine effect, inducing angiogenesis, anti-apoptosis, anti-inflammatory, immunoregulation, autophagy regulation, antifibrosis, anti-oxidative stress and mitochondrial transfer.

Studies have shown that BMSCs can improve ovarian reserve function in POF patients through various mechanisms, including homing, paracrine, regulation of ovarian angiogenesis, anti-fibrosis, anti-inflammatory and immune regulation, anti-apoptosis, mitochondrial transfer, autophagy regulation (**Figure 2**, **Table 1**).

**Table 1:** Therapeutic effects of MSCs in POF.

Cell type	Delivery method	Model	Main effects	Reference
BMSCs	Intraovarian injection	CTX-induced POF	Homing to injured ovary. Decreased GCs apoptosis.	Sameni HR et al., 2019 (48)
BMSCs	intravenous injection	Radiotherapy-induced POF	Regulated apoptosis, proliferation and differentiation of ovarian follicles through genetic and epigenetic modulation of the integrated TGF-β, Wnt/β-catenin, and Hippo pathways.	El-Derany MO et al., 2021 (13)
BMSCs	intravenous injection	CTX-induced POF	Recovered normal serum hormonal levels. Promoted formation of primordial follicles.	Badawy A et al., 2017 (14)
BMSCs	N/A	phosphoramide mustard induced GCs injury	Reduced the senescence and apoptosis of GCs. Reduced cleaved-Caspase 3 expression.	Chen S et al., 2020 (21)
BMSCs	Intraovarian injection	CTX-induced POF	Inhibited GCs apoptosis. Ameliorated hormone level and ovarian function.	Chen X et al., 2018 (27)
BMSCs	Intraperitoneal injection	CTX-induced POF	Increased the number of healthy follicles. Restored estrous cycle and ovarian function. Inhibited GCs apoptosis.	Yang M et al., 2020 (37)
BMSCs	Intraovarian injection	CTX and BUS-induced POF	Restored ovarian hormone production. Reactivated folliculogenesis. Improving pregnancy outcomes.	Mohamed SA et al., 2018 (49)
BMSCs	Intraovarian injection	CTX-induced POF	Regulated Bcl-2 and Bax expression. Restored ovarian function.	Zarbakhsh S et al., 2019 (50)
BMSCs	intravenous injection	CTX-induced POF	Differentiation into specific cellular phenotypes. Secreted VEGF and decreased GCs apoptosis. Recovered normal serum hormonal levels.	Abd-Allah SH et al., 2013 (51)
BMSCs	intravenous injection	CTX-induced POF	Homing to the stroma of the injured ovaries. Ameliorated hormonal level, folliculogenesis and ovarian architecture. Restored ovarian function *via* regulating microenvironment surrounding the oocytes (TNF-α, IGF1).	Gabr H et al., 2016 (52)
BMSCs	intraovarian injection	CTX and BUS-induced POF	Restored fertility in POF mouse. Engrafted BMSCs didn’t differentiate to replace ovarian cells. Restored ovarian GCs by paracrine effect.	Park HS et al., 2021 (53)
BMSCs	Intraperitoneal injection	CTX-induced POF	Increased primordial follicle counts and AMH levels.	Besikcioglu HE et al., 2019 (54)
BMSCs	intravenous injection	CTX and BUS-induced POF	Decreased Bax, p53, p21 and increased CyclinD2 expression. Inhibited apoptosis. Promoted residual ovarian cell proliferation.	Bao R et al., 2018 (55)
BMSCs	intravenous injection	Cisplatin- induced POF	Homing to injured ovaries. Restored ovarian function and structure. Increased E2 levels and follicle numbers.	Liu J et al., 2014 (56)
BMSCs	intraovarian injection	CTX-induced POF	Improved ovarian function and structure mainly by paracrine effect	Khanmohammadi N et al., 2018 (57)
BMSCs	intraovarian injection	CTX and BUS-induced POF	Stimulated GCs proliferation and inhibited apoptosis. Stimulated E2 production. Restored ovarian structure and function *via* paracrine effect.	Park HS et al., 2021 (58)
BMSCs	intraovarian injection	N/A	Diminished menopausal symptoms. Resumed menses. Increased E2 levels.	Igboeli P et al., 2020 (59)
BMSCs	N/A	CTX-induced POF	Homing to the ovarian tissue. Protected germ cells from cyclophosphamide-induced cell apoptosis and DNA damage.	Kilic S et al., 2014 (60)
BMSCs	intraovarian injection	CTX-induced POF	Reduced GCs apoptosis and induced up-regulation of Bcl-2. Released VEGF, HGF and IGF-1.	Fu X et al., 2008 (61)
BMSCs	intravenous injection	CTX and BUS-induced POF	Increased follicles numbers, decreased GCs apoptosis and restored FSH and E2 levels by releasing cytokine (VEGF, HGF, IGF-2).	Bahrehbar K et al., 2020 (62)
BMSCs	intravenous injection	D-galactose induced aging rat model	Homed to ovarian tissue. Affected the content of MDA and activity of SOD, and improve the aging of reproductive organs by reducing p16 expression and increasing PCNA expression.	Wang Z et al., 2020 (63)
ADMSCs	intravenous injection	CTX-induced POF	Reduced ovarian injuries. Inhibited GCs apoptosis and proapoptotic protein expression. Promoted antiapoptotic protein expression (VEGF and VEGFR2). Migration and homing to ovary mediated by SDF-1/CXCR4 axis *via* PI3K/Akt signaling pathway.	Ling L et al., 2022 (64)
ADMSCs	intravenous injection	CTX-induced POF	Improved ovarian function partly through paracrine mechanism (FGF2, IGF-1, HGF, VEGF). Inhibited GCs apoptosis. Promoted angiogenesis. Regulated follicular development.	Ling L et al., 2019 (65)
PDMSCs	intravenous injection	Ovariectomized rat model	Restored ovarian function *via* activating PI3K/AKT signaling pathway and increasing VEGF expression and thereby promoting vascular remodeling.	Cho J et al., 2021 (66)
PDMSCs	intravenous injection	zona pellucida glycoprotein 3 induced autoimmune POF	Reduced GCs apoptosis induced by endoplasmic reticulum stress-related inositol-requiring enzyme 1α signaling pathway.	Li H et al., 2019 (67)
PDMSCs	intravenous injection	Ovariectomized Rat Model	Homing to damaged ovarian tissue. Restored ovarian function *via* upregulating antioxidant factors (SOD, catalase).	Seok J et al., 2020 (68)
MenSCs	intravenous injection	Cisplatin induced POF	Ameliorated ovarian fibrosis. Increased follicles numbers. Decreased GCs apoptosis. Normalized hormone levels. Improved ovarian function *via* paracrine mechanism by secreting FGF2.	Wang Z et al., 2017 (69)
UMSCs	intravenous injection	Cisplatin induced POF	Restored ovarian function and alleviated theca interstitial cells apoptosis by regulating autophagy signaling pathway AMPK/mTOR.	Lu X et al., 2020 (70)

ADMSCs, amnion-derived mesenchymal stem cells; BUS, busulfan; CTX, cyclophosphamide; CM, conditioned medium; CXCR4, C-X-C chemokine receptor 4; E2, estradiol; FGF2, fibroblast growth factor2; FSH, follicle-stimulating hormone; GCs, granulosa cells; HGF, hepatocyte growth factor; IGF, insulin-like growth factor; MDA, malondialdehyde; MenSCs, menstrual-derived stem cells; PDMSCs, placenta-derived mesenchymal stem cells; POF, premature ovarian failure; PCNA, proliferating cell nuclear antigen; SOD, superoxide dismutase; SDF1, stromal-derived factor1; TNF-α, necrosis factor alpha; TGF-β, transforming growth factor-β. UMSCs, umbilical cord-derived mesenchymal stem cells; VEGF, vascular endothelial growth factor; VEGFR2, vascular endothelial growth factor receptor2.

### The homing of BMSCs

**Figure 3:**
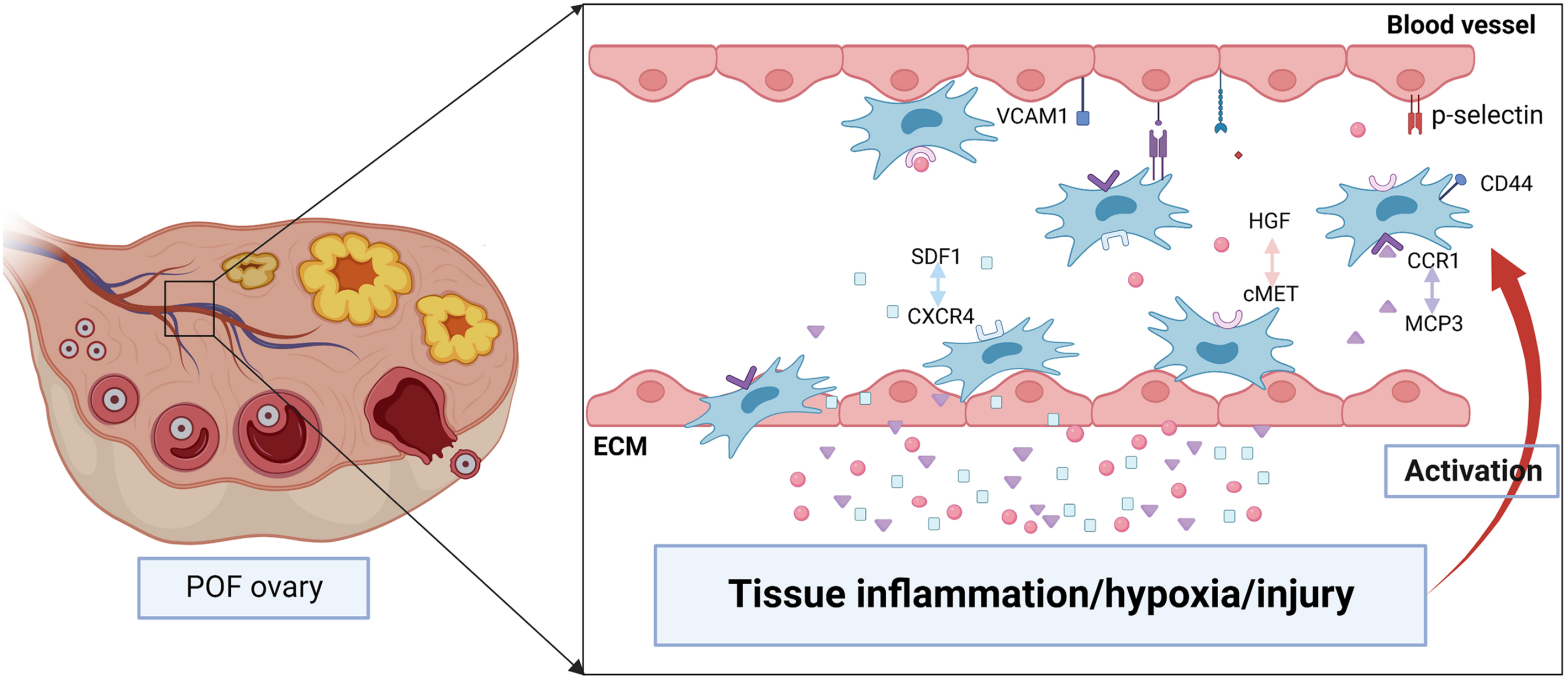
The possible mechanisms of BMSCs homing to injured ovary. Tissue inflammation, hypoxia or injury may induce the high level of chemokines including stromal cell derived factor 1 (SDF1), hepatocyte growth factor (HGF), monocyte chemotactic protein-3 (MCP3). They are released into the bloodstream and promote BMSCs proliferation as well as express specific receptors such as CXC chemokine receptor 4 (CXCR4), cMET, CC chemokine receptor 1(CCR1). After binding with the ligand, BMSCs migrate to the injured tissue along the gradient of chemokines concentration. In addition, various adhesion molecules including CD44, p-selectin, vascular cell adhesion molecule 1 (VCAM1) participate in regulating the homing of BMSCs.

MSCs homing is the process that self-derived or exogenous MSCs are captured in the vasculature of the target tissue and then migrate to the target tissue across vascular endothelial cells actuated by a variety of factors, which will undergo the process of selectin thrombus, cytokine activation, integrin block, transvascular endothelial cell and extravascular migration towards chemokine gradient (71, 72). Lu J et al. indicated that the therapeutic effect of BMSCs was evaluated through the amount of BMSCs migration to the site of lesion (73). Therefore, it’s crucial to understand the detailed mechanism about the homing of BMSCs (**Figure 3**).

The homing of stem cells is regulated by “stem cell niches microenvironment” of target tissues, which regulates the proliferation, migration and differentiation of stem cells through different signaling pathways. A large number of signaling molecules including stromal cell derived factor1(SDF1), hepatocyte growth factor (HGF), monocyte chemotactic protein (MCP)3, platelet-derived growth factor are released in injured tissues, while these factors stimulate high expression of specific receptors (CXC chemokine receptor 4 (CXCR4), cMET, CC chemokine receptor 1, platelet-derived growth factor receptor, respectively) on the surface of BMSCs, thereby promoting the homing of BMSCs (74, 75). Chemokines are closely relevant to activation and migration of cell, and at the same time, play a key role in many diseases including hematopoiesis, immune monitoring and inflammation, morphogenesis and neovascularization, as well as autoimmune diseases and cancer. Chemokine CXCL12, also known as SDF1, is one of the important molecules that induce homing of stem cells (76). The subtypes of CXCL12 are various, among which SDF1α and SDF1β are the main forms. SDF1 is widely expressed in many tissues including brain, thymus, heart, lung, liver, kidney, spleen and bone marrow, and maintains low secretion physiologically (77). It can activate phosphatidylinositol-3-kinase (PI3K)/Akt or mitogen-activated protein kinase (MAPK)/extracellular signal-related kinas (ERK) signaling pathway by binding to specific receptors and then regulate cell migration, proliferation, apoptosis, tube formation and chemotaxis (78). The receptors of SDF1 are various, among which CXCR4 is the typical one. CXCR4 is a G-protein-coupled receptor. SDF1/CXCR4 is involved in cell migration in early embryonic development and may influence stem cell migration from bone marrow or niche to damaged tissue during the whole life, especially in adulthood (79). Studies have shown that SDF1/CXCR4 axis plays a key role in promoting MSCs homing and survival (80). First, when contacting with CXCR4 at the extracellular domain, SDF1 will induce conformational change of the receptor, and which will strengthen SDF1 binding with the receptor pocket (81). Then, CXCR4 experiences a second conformational change, which activates the intracellular trimeric G protein by dissociating of Gα subunit from the Gβ/Gγ dimer (81). Activated G proteins can activate multiple signal pathways, and then participate in increasing intracellular calcium, modifying cellular proteins, altering transcription factor binding and gene expression, thereby regulating cell proliferation, migration, survival and senescence (81). The expression of SDF1 is greatly increased in damaged tissues, which promotes the homing and survival of stem cells in the damaged tissue respectively by binding with CXCR4 and CXC chemokine receptor 7 on the surface of MSCs (82). Studies have shown that BMSCs mobilized from bone marrow to peripheral blood and then migrated to injured tissue, possibly along with the gradient of SDF1 concentration (83). Tamari et al. found that adding SDF-1 in standard medium could promote the migration of MSCs, while the MSCs migration was significantly reduced after the intervention of SDF1 receptor antagonist (84). Similarly, after transducing lentivirus carrying SDF1α into mouse BMSCs, overexpression of SDF1α can promote the proliferation, migration, and osteogenic differentiation of BMSCs, and which partly by activating the Wnt pathway (85). In addition, when lentivirus carrying CXCR4 are transduced into human BMSCs, the migration ability of CXCR4-BMSCs toward SDF1 is significantly increased due to the overexpression of CXCR4 (86). Ling L et al. also indicated that the levels of SDF1 in ovaries and serum were remarkably increased in rats with cyclophosphamide-induced POF, and ovaries with POF induced the homing of MSCs expressing CXCR4 (64), which further confirmed that SDF1/CXCR4 axis partially regulated the migration and homing of transplanted MSCs to the ovaries of POF.

HGF is a growth factor consisting of α and β chains, which contains four cyclic domains and one serine protease-like domain, respectively. Evidences demonstrate that HGF plays an important role in growth stimulation, tissue regeneration, migration, angiogenesis, morphogenesis, tumorigenesis, and tumor invasion (87). In addition, Han P et al. found that HGF could promote the proliferation and differentiation of pluripotent stem cells, ESCs and BMSCs (88). The ovulation process is thought to be hormone-induced tissue injury (89). HGF is activated through the process of ovulation injury-tissue factor–thrombin–HGF activator–HGF cleavage and promote repair of tissue injury after ovulation (90), which indicates that ovarian injury increases the level of HGF. Han P et al. also confirmed that the expression of HGF in injured tissues was significantly increased (88). The receptor tyrosine kinase cMET is the specific receptor of HGF, which is a member of the transmembrane tyrosine kinase receptor superfamily and has independent phosphorylation activity (91). The high-affinity binding of HGF to cMET induces homodimerization and autophosphorylation of the cytoplasmic domain of cMET, and then activates HGF/cMET and downstream pathways such as the MAPK/ERK, PI3K, p-38, and the Akt/protein kinase B pathways, thereby promoting cell proliferation, invasion, survival, motility and angiogenesis (88, 92, 93). The HGF/cMET pathway plays an important role in the BMSCs homing. Studies have shown that the high level of HGF in injured tissues can upregulate the expression of cMET in stem cells (88). And the overexpression of cMET promotes the homing of BMSCs significantly (94).

MCP is a member of the CC chemokine family, which mediates cell chemotactic. Previous studies demonstrated that significant upregulation of the stem cell homing cytokine MCP-3 in urethral and vaginal tissues following simulated birth trauma (95–97). Yamada et al. found that MCP-1 and MCP-3 were the homing factors of MSCs, which could recruit MSCs to the injured tissues (98, 99). Cui L et al. indicated that the level of TGF-β1 in ovarian tissue of POF rats was increased (100). It can promote BMSCs migrate to lesion sites *in vitro* and *in vivo* though histone demethylase KDM6B mediated inhibition of methylation marker H3K27me3 (101). Clinical studies also showed that the level of chemokines and growth factors of POF patients in follicular fluid significantly increased comparing with control group, including interferon-γ-inducible protein 10, macrophage inflammatory protein-1α, C-X-C motif chemokine ligand 8, eosinophil chemokine factor-1 and leukaemia inhibitory factor as well as brain-derived neurotrophic factor, vascular endothelial growth factors (VEGF)‐D and basic fibroblast growth factor (bFGF) (102), which may be tightly related to enhance the homing efficiency of BMSCs.

In the process of chemotaxis to damaged tissue, apart from the chemokines mentioned above, the rolling and adhesion process of MSCs are also regulated by various adhesion molecules including CD44, vascular cell adhesion molecule 1, intercellular adhesion molecule 1, p-selective protein, integrin and α4β1 (103). Finally, MSCs cross vascular endothelial cells and basement membrane under the mediation of matrix metalloproteinases and follow the gradient of chemokine concentration homing to the target tissues (104).

Recent studies have confirmed that BMSCs can migrate to damaged parts and ameliorate ovarian structure and function *via* inhibiting apoptosis, promoting proliferation and improving folliculogenesis in POF mice (13, 49, 50). Moreover, BMSCs regulates the function of local cells after homing through intercellular contact (105). Previous studies have shown that BMSCs can differentiate into specific cells to replace damaged cells and then repair damaged tissues (51, 106, 107). On the contrary, recent studies indicated that the transplanted BMSCs were located in the interstitial area rather than in follicles of ovary, which suggested that BMSCs could homing to the injured ovary and may enhance the ovarian function by regulating the microenvironment around ovarian follicles rather than differentiating into oocytes or GCs (49, 52). Park HS et al. also confirmed that the engrafted BMSCs to the POF ovary didn’t differentiate into ovarian cells, but restored ovarian GCs *via* secreting paracrine factors (53). Moreover, the number of engrafted BMSCs decreased gradually within 2 weeks and disappeared entirely in majority animals within 4 weeks after transplantation (53). It is remarkable that the number of transplanted stem cells in injured tissues isn’t always correlated with the healing rate, because even though stem cells can’t exist in the target tissues for a long time, their paracrine and autocrine roles may help to heal and activate local stem cells of stationary stage, which may promote the recovery of damaged tissue (54). Zahra et al. also indicated that there were small number of OGSCs in ovarian surface epithelium and cortical tissues, which could express ovarian germline markers and differentiate into cells of all three embryonic germ layers (108). And in addition, these cells could generate new GCs and primary follicles (108). Therefore, it can be concluded that BMSCs may restore the structure and function of damaged ovary by activating OGSCs (109), which can differentiate into new GCs and primary follicles, but the specific mechanism still needs to be further studied.

It isn’t completely clear that whether or not BMSCs can differentiate into ovarian cells after homing to injured ovary. Currently, it is widely believed that the key of BMSCs restoring POF is based on paracrine effect of stem cells rather than differentiation. However, it is remarkable that these secretory factors are not the only mechanism that BMSCs improve ovarian function. Ling et al. demonstrated that MSCs transplantation was more effective in alleviating ovarian damage and restoring ovarian function in POF rats compared with injection of MSCs conditioned media (CM) (65). Therefore, further studies are needed to clarify the mechanism of interaction between BMSCs and ovaries, which is greatly significant to the clinical development of BMSCs transplantation.

### Paracrine effect of BMSCs

BMSCs can synthesize and secrete a variety of chemokines, growth factors and hormones, including VEGF, insulin-like growth factor-1 (IGF-1), HGF, bFGF (50, 55). These molecules play important roles in angiogenesis, anti-fibrosis, anti-inflammatory and immunomodulatory, anti-apoptotic, thereby improving the local microenvironment and promoting the recovery of damaged tissues (**Table 2**). Many studies have shown that BMSCs restore ovarian structure and function possibly through paracrine effect (49, 53, 56). Among all the BMSCs paracrine factors, brain-derived neurotrophic factor is a member of the neurotrophic factor growth factor family, which promotes oocyte maturation and embryo development (102). bFGF and VEGF are involved in ovarian angiogenesis, which help to provide nutrition for GCs (102). Meanwhile, VEGF and its receptors play an important role in inhibiting apoptosis of GCs, and promoting the development of follicles (51, 110, 111). IGF-1 is a growth hormone that stimulates GCs proliferation by regulating DNA replication in theca cells and GCs, and helps to enhance the function of gonadotropin, regulate the activity of aromatase and promote the formation of follicular cavity as well as inhibit cell apoptosis (48). HGF has significant anti-apoptosis effect in ovarian GCs and oocytes, which helps to promote blood vessel growth and improve ovarian function (113). bFGF serves as an initiator of folliculogenesis *via* inducing primordial follicle development (117). Although BMSCs transplantation has been widely used in repairing the damaged tissues, it has been reported that there are some risks in stem cells transplantation including tumor formation, pulmonary embolism (122, 123).

**Table 2:** The roles of paracrine factors derived from MSCs.

Factors	Function	Reference
bFGF, CCL11, HGF, IGF1, IL-6, IL8, MCP1, M-CSF, metalloproteinase 3, TGF-α, VEGF	Promoted angiogenesis	Liu P et al.2020 (102), Gabr H et al., 2016 (52), Yao X et al.2016 (110), Rühle A et al., 2019 (111), Lin S et al., 2017 (112), Zhang S et al., 2020 (113), Maacha S et al., 2020 (114), Park HS et al., 2019 (115)
BDNF	Promoted oocyte maturation and embryo development	Liu P et al.2020( (102)
SDF1	Promoted BMSCs secrete VEGF and differentiate into vascular endothelial cells.	Fang J et al., 2021 (116)
bFGF, VEGF	Inhibited GCs apoptosis and promoted the development of follicles.	Yao X et al., 2016 (110), Rühle A et al., 2019 (111), Wang L et al., 2013 (117)
IGF1	Enhanced the function of gonadotropin. Regulated the activity of aromatase. Promoted the formation of follicular cavity. Inhibited cell apoptosis	Sameni HR et al., 2019 (48)
IL-10, HO-1, HGF, IDO, NO, PGE2, TGF-β	Inhibited inflammatory response. Immunomodulatory.	Zhang L et al., 2019 (118), Forsberg MH et al., 2020 (119)
cyclophilin A, DJ-1 cyclophilin B, IGF1, thioredoxin, HSP27, peroxiredoxin-1	Anti-oxidative stress effect.	Gabr H et al., 2016 (52), Pires AO et al., 2016 (120)
HO-1	Regulated autophagy *via* activating JNK/Bcl-2 signaling pathway. Upregulated the number of CD8+CD28− T cells in the circulation.	Yin N et al., 2020 (121)

bFGF, basic fibroblast growth factor; CCL11, C-C motif chemokine ligand 11; GCs, granulosa cells; HO-1, heme oxygenase 1; HGF, hepatocyte growth factor; HSP, heat shock protein; IDO, indoleamine 2,3 dioxygenase; MCP1, monocyte chemoattractant protein1; M-CSF, macrophage colony-stimulating factor; NO, nitric oxide; PGE2, prostaglandin E2; SDF1, stromal cell-derived factor-1; TGF-β, transforming growth factor-beta; TGF-α, transforming growth factor-α.

In order to find the safer treatments, the researcher study on whether the CM of BMSCs is a feasible treatment. BMSCs-CM contains a variety of cytokines, such as VEGF, HGF, IGF-1 etc., which can inhibit apoptosis and promote proliferation of GCs *in vivo* or *in vitro*. These results indicate that the secretion of these factors plays an important role in BMSCs improving ovarian function (124). Khanmohammadi N.et al. indicated that injecting the BMSCs-derived CM or BMSCs into the damaged ovaries had almost the same effect on repairing the damaged ovaries (57). Similarly, human BMSCs-derived CM plays a similar role to human BMSCs in reducing apoptosis of human GCs, promoting cell proliferation, improving the viability of ovarian GCs and restoring ovarian structure as well as stimulating estrogen production (58). Above studies indicated that paracrine effect plays a major role in BMSCs therapy. However, Ling et al. indicated that the therapeutic effect of MSCs medium on POF was not as good as MSCs (65). Thus, whether the efficiency of BMSCs-CM is equal to BMSCs still warrants further study.

### Angiogenesis

**Figure 4:**
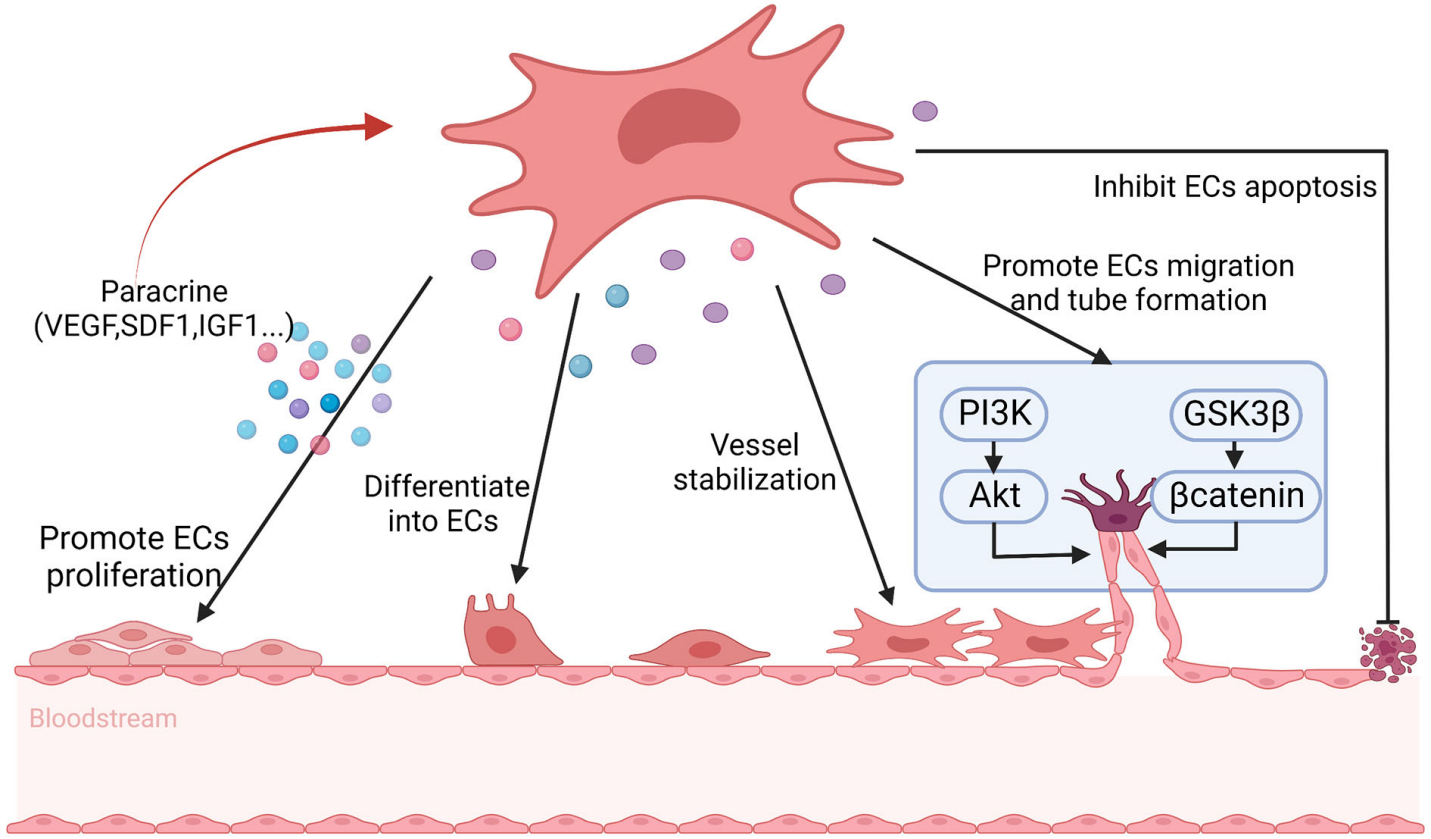
The possible mechanisms of BMSCs promote angiogenesis in POF. BMSCs help to promote angiogenesis through differentiating into endothelial cells (ECs), maintaining vascular stability, inhibiting ECs apoptosis and secreting angiogenic factors, including vascular endothelial growth factor (VEGF), stromal cell derived factor 1 (SDF1) and insulin-like growth factor 1 (IGF1) et. to promote ECs proliferation, as well as promoting ECs migration and tube formation *via* PI3K/Akt and GSK3β/βcatenin signal pathway. At the same time, the secrete factors reinforce the angiogenesis of BMSCs in turn.

Angiogenesis plays an important role in repairing damaged ovaries. Previous studies have shown that MSCs may participate in angiogenesis through the following two mechanisms. Firstly, MSCs have the potential to differentiate into endothelial cells, vascular smooth muscle cells and other types of cells. Secondly, MSCs can secrete a variety of bioactive factors that can promote angiogenesis (125). Comparing with other tissue-derived MSCs, BMSCs have higher angiogenic activity, which can differentiate into endothelial cells, pericytes, and vascular wall, and then promote angiogenesis (59). Moreover, BMSCs contributing to angiogenesis at least partly depending on secreting various angiogenic factors including VEGF, MCP1, interleukin-6, SDF-1α, macrophage colony-stimulating factor, IL-1 receptor, IGF-1, interleukin-8, metalloproteinase 3 (114). The composition and concentration of angiogenic factors will also ultimately affect the functional responses of BMSCs (114). VEGF is a powerful angiogenic factor and significantly affects ovarian angiogenesis, which is closely related to follicular formation and development (110, 111). After binding to the receptor, VEGF activates endogenous VEGF signaling through the PI3K/Akt and GSK3β/β-catenin pathways, which results in ovarian vascular remodeling and ultimately enhances follicular formation (66). IGF-1 is also an effective angiogenic factor, which is highly expressed in the damaged vessels (52). After binding to its receptors, IGF-1 can activate the PI3K/Akt signaling pathway, which induces endothelial cell proliferation, differentiation and migration, and then possibly improves the structure and function of damaged ovaries by promoting angiogenesis (52, 112). SDF1 secreting by BMSCs can directly promote the differentiation of BMSCs into vascular endothelial cells through binding to CXCR4 on BMSCs (116). In addition, SDF1 indirectly promotes the proliferation of vascular endothelial cells by promoting the secretion of VEGF from BMSCs (116). BMSCs-derived CM treatment can activate human ovarian vascular endothelial cells, and then in which the expression of angiogenic marker genes, transforming growth factor-α, C-C motif chemokine ligand 11 are increased, which inducing endothelial cell proliferation and promoting angiogenesis as well as increasing the density of new blood vessels (115). Moreover, studies have shown that the key mechanism of BMSCs CM enhancing angiogenesis is highly correlated with the PI3K/Akt pathway (115). Zhang et al. also found that BMSCs may control angiogenesis and follicular survival of xenografted human ovarian tissues by angiotensin (126) (**Figure 4**).

### Anti-fibrosis effect

In ovarian tissue, stromal cells can proliferate and differentiate into endometrial cells or myofibroblasts. The myofibroblasts synthesize and secrete extracellular matrix including type I and III collagen fibers. The excessive accumulation of extracellular matrix will lead to organ fibrosis, which is the basic pathological change of POF (100). Ovarian fibrogenesis is associated with various cytokines, including matrix metalloproteinases, tissue inhibitors of metalloproteinases, transforming growth factor-β1 (TGF-β1), connective tissue growth factor, peroxisome proliferator-activated receptor γ, VEGF, endothelin -1 (127). TGF-β1 is a key mediator of tissue fibrosis, which is involved in fibrosis of multiple organs by activating its downstream small mother against decapentaplegic (Smad) signaling and triggering pre-fibrotic gene overexpression (128). Moreover, Inagaki Y et al. also indicated that TGF-β participated in inducing transcription of alpha-smooth muscle actin and other extracellular matrix proteins (129), which would promote the development of tissue fibrosis. Studies confirmed that the level of TGF-β1 in ovarian tissue of POF rats was increased notably (100). And after BMSCs transplantation, the level of TGF-β1 in the POF ovary was down-regulated prominently (13). Moreover, Cui et al. found that human umbilical cord-derived MSCs (hUMSCs) inhibited ovarian fibrosis by regulating stromal cell differentiation through TGF-β1/Smad3 signaling pathway, thereby promoting the recovery of ovarian function in POF rats (100). And MSCs derived from menstrual blood help to improve ovarian function by reducing ovarian interstitial fibrosis and apoptosis in GCs, which may be partially mediated by secreting fibroblast growth factor 2 (69). Therefore, we guess that BMSCs may also inhibit ovarian fibrosis by the same mechanism. However, due to the reactivity of different sources of MSCs is diverse, further studies are needed to confirm whether this hypothesis is reliable. In addition, Wang PP et al. indicated that the anti-fibrosis effect of BMSCs in liver fibrosis induced by lipopolysaccharide was through secreting HGF and cell-cell contact, which was closely bound up with the inhibition of Toll-like receptor 4/Myeloid differentiation primary response gene 88/nuclear factor kappa-B signaling pathway (130). In view of this, whether BMSCs protect ovarian fibrosis in POF model (131) by the same mechanism is worthy of further exploration.

Chronic inflammation is one of the main factors driving the process of fibrosis, which can change the normal structure of tissues and result in functional deterioration. BMSCs exhibit significant anti-inflammatory bioactivity, which may be another mechanism of BMSCs inhibiting ovarian fibrosis. BMSCs promote the secretion of anti-inflammatory cytokine IL-10 and inhibit the expression of pro-inflammatory cytokines tumor necrosis factor α and interleukin-6, thereby inhibiting the inflammatory response, which plays an important role in anti-fibrosis effect (118). In addition, angiogenesis and reperfusion are also critical to repair damaged tissue and prevent fibrosis.

### Anti-inflammatory and immunomodulatory effects

**Figure 5:**
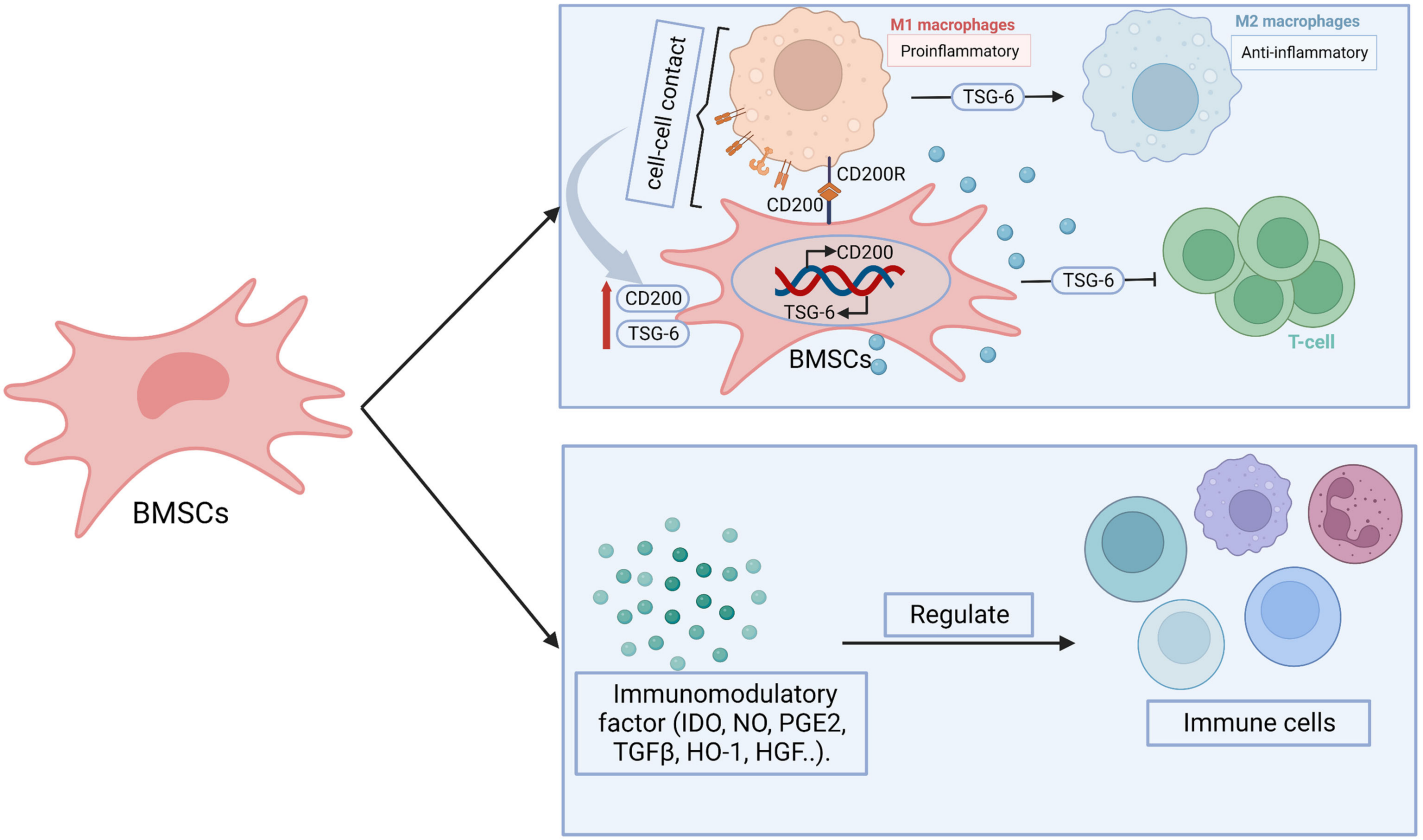
The possible mechanisms of BMSCs in immunoregulation. BMSCs display immunomodulatory effect through paracrine effect and cell-cell contact. BMSCs secrete immunomodulatory related factors, including indoleamine 2,3 dioxygenase (IDO), nitric oxide (NO), prostaglandin E2 (PGE2), transforming growth factor-β (TGF-β), heme oxygenase 1 (HO1), hepatocyte growth factor (HGF), which participate in regulating the number or function of immune cells. BMSCs-derived tumor necrosis factor-stimulated gene-6 (TSG-6) promote the transform from pro-inflammatory macrophages (M1) to anti-inflammatory (M2) phenotype as well as inhibit the proliferation and inflammatory response of T cells. The paracrine effect is heightened after BMSCs- M1 contact, which enhance the expression of TSG-6. Moreover, CD200R on M1 bind with CD200 on BMSCs promote the transition of M1 to M2. At the same time, which contribute to promote the expression of CD200 on BMSCs.

Abnormally elevated levels of chemokines and cytokines in follicular fluid of POF patients will induce intracellular inflammatory in follicular niche by cellular and paracrine interactions, which adversely affects oocyte quality and the function of GCs or theca cell (102). Moreover, it can attract a large number of white blood cells to migrate to the ovaries or activate plenty of immune cells, which leads to chronic low-grade inflammatory state, all of which will further aggravate follicular atresia and apoptosis, and ultimately affect the quality and quantity of oocytes (102). The concentrations of pro-inflammatory cytokines interleukin-6, interleukin-8 and tumor necrosis factor α in serum of chemotherapy-induced POF mice were significantly increased, while the levels of anti-inflammatory cytokines IL-10 were markedly decreased (132). The abnormalities of inflammation-related factors may result in apoptosis of GCs, which tightly contributed to the development of ovarian damage in POF mice (132). The signaling molecules from inflammatory sites promote the homing of BMSCs, which can help to inhibit the production of inflammatory cytokines and proliferation of lymphocytes, thereby inhibiting local inflammation (133). The immunomodulatory function of MSCs is mainly through intercellular contact, paracrine activity and interaction with T cells, B cells, natural killer cells, macrophages, monocytes, dendritic cells and neutrophils (134) (**Figure 5**). For example, the direct contact betwixt the proinflammatory macrophages and BMSCs promotes not only the production of tumor necrosis factor-stimulated gene-6, but also the expression of CD200 on BMSCs (105). The elevated tumor necrosis factor-stimulated gene-6 helps to suppress the proliferation of T cells and promote the transform between proinflammatory macrophages and anti-inflammatory phenotype, while the increased CD200 participates in mediating the interaction betwixt BMSCs and proinflammatory macrophages (105). Moreover, BMSCs secrete a variety of immunomodulatory mediators, such as indoleamine 2,3 dioxygenase, nitric oxide, prostaglandin E2, TGF-β, heme oxygenase 1, HGF, which play an important role in anti-inflammatory and immunomodulatory (119). Pro-inflammatory cytokine interferon-γ has a synergistic immunosuppressive effect with BMSCs, which mediates immune regulation by upregulating the expression of prostaglandin E2, HGF, TGF-β1 in BMSCs and inducing the expression of indoleamine 2,3 dioxygenase as well as participating in tryptophan catabolism (135). Regulatory T cells deficiency is associated with the pathogenesis of POF *via* mediating apoptosis and steroidogenesis dysfunction of GCs (136). Luz-Crawford P et al. found that BMSCs were capable to induce functional regulatory T cells during the differentiation process of Th1 and Th17 cells, which was related to the increase of IL-10 production by BMSCs (137). Moreover, BMSCs participate in regulating the number and function of T cells (138). Therefore, we surmise that maybe BMSCs regulate immune system in POF through procedure mentioned above.

### Anti-apoptotic effect

**Figure 6:**
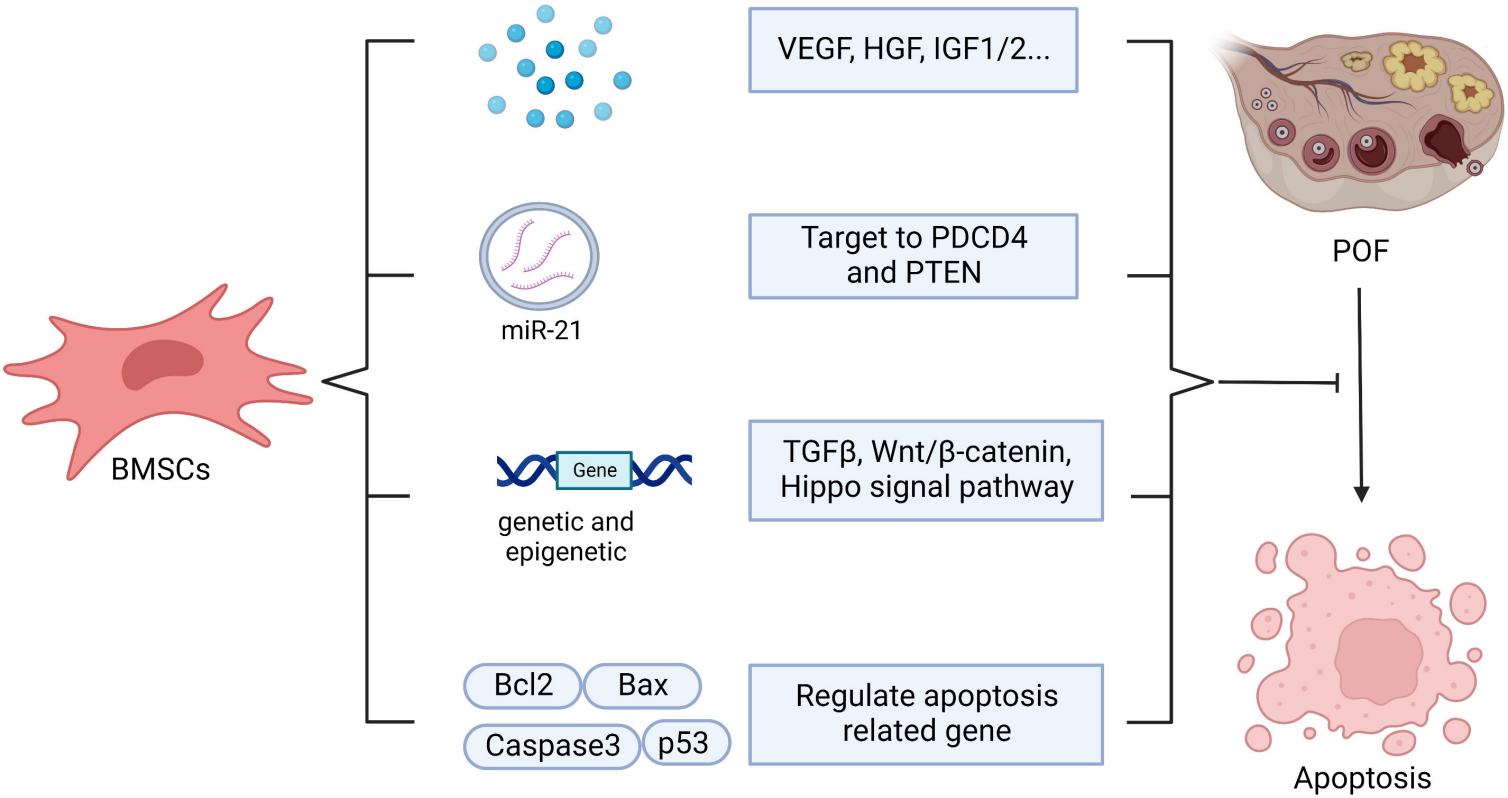
The possible mechanisms of BMSCs in anti-apoptosis. BMSCs help to inhibit apoptosis through secreting vesicle containing microRNA-21(miR-210), which targeting to PDCD4 and PTEN, or secreting vascular endothelial growth factor (VEGF), hepatocyte growth factor (HGF), insulin-like growth factor 1/2 (IGF1/2) or and regulating apoptosis related gene, including Bcl2, Bax, Caspase3, p53, as well as regulating genetic and epigenetic *via* TGFβ, Wnt/β-catenin, Hippo signal pathway.

Apoptosis of GCs and theca cells increase in the ovaries of cyclophosphamide (CTX)-induced POF mice (55). BMSCs transplantation can help to inhibit the apoptosis of ovarian cells by regulating the levels of apoptosis-related genes such as Bax, p53, caspase-3 and Bcl-2. Meanwhile, it can regulate the expression of cyclinD2 and p21 to promote the proliferation of residual ovarian cells, so as to repair the structure and function of damaged ovary (37, 55). Kilic S et al. revealed that BMSCs could protect germ cells from CTX-induced cell apoptosis and DNA damage (60). In addition, BMSCs regulate apoptosis, proliferation, and differentiation of ovarian follicles *via* genetic and epigenetic regulation of the integrated TGF-β, Wnt/β-catenin and Hippo signaling pathways, which are associated with ovarian follicles growth and maturation (13). Transplanting BMSCs with overexpressing miR-21 into the rat ovaries damaged by chemotherapy can more efficiently inhibit the apoptosis of GCs and improve the ovarian structure and function through targeting to PDCD4 and PTEN (the target genes of miR-21) compared with the transplantation of BMSCs or miR-21 alone (139). In addition, BMSCs transplantation may inhibit GCs apoptosis and ameliorate ovarian function through releasing VEGF, HGF, IGF-1 and IGF-2, while upregulating the expression of Bcl-2 (61, 62). Endoplasmic reticulum stress plays a crucial role in promoting autophagy and apoptosis in GCs, which results in excessive follicle loss and endocrine disorders (67, 140). Hongxing Li et al. found that human placenta MSCs (hPMSCs) transplantation inhibited the activation of inositol-requiring enzyme 1α pathway of endoplasmic reticulum stress in ovaries and reduced the up-regulation of XBP1, GRP78, caspase-12 in zona pellucida glycoprotein 3 peptides-induced POF mice, which subsequently decreased GCs apoptosis (67). Hence, it’s worth to explore whether BMSCs restore ovarian function through regulating endoplasmic reticulum stress as hPMSCs (**Figure 6**).

### Antioxidation

Oxidative stress participates in inducing lipid peroxidation functionally and structurally, and changing protein and DNA as well as promoting apoptosis, which plays an important role in the pathogenesis of POF (17, 141). Ağaçayak E et al. found that the total oxidation status and oxidative stress index levels were increased in POF patients (17). Superoxide dismutase (SOD), which is an antioxidant enzyme, can help to partially restore ovarian function by inhibiting ROS production (141). However, the levels of SOD and nuclear factor erythroid 2-related factor (Nrf2) were decreased in the POF mice ovary, leading to the accumulation of ROS (142). ROS accumulation may impair to ovarian function and oocyte quality (141). Several studies have found that BMSCs play an important role in antioxidant stress. Proteomic analysis revealed that various antioxidant mediators such as cyclophilin A, cyclophilin B, thioredoxin, DJ-1, heat shock protein 27, peroxiredoxin-1 secreted by BMSCs showed significant antioxidant stress effects (120). IGF1 releasing from BMSCs also possesses effective anti-oxidative abilities (52). In addition, BMSCs transplantation impacts the activity of SOD and the content of malondialdehyde through reducing the expression of cyclin-dependent kinase inhibitor 2A (P16) and increasing proliferating cell nuclear antigen, thus improving the morphology and function of ovary (63). Seok J et al. indicated that the transplanted hPMSCs reduced oxidative stress and apoptosis in ovariectomized rat model *via* changing the expression of HO-1/HO-2 and enhancing catalase and SOD1 gene expression (68). Furthermore, hUMSCs improve cisplatin-induced autophagy in injured ovarian tissue *via* reducing the levels of ROS and regulating AMPK/mTOR signaling pathway (70). From this, we conjecture that maybe the mechanism of BMSCs in anti-oxidative stress is consistent with aforesaid MSCs. Currently, there are few studies about the mechanism of BMSCs regulating oxidative stress to improve POF ovarian function, but they have been widely studied in other diseases. Niu Y et al. revealed that BMSCs-CM could alleviate oxidative stress injury of neural stem cells through decreasing the expression of lactate dehydrogenase and malondialdehyde, increasing the expression of SOD and inhibiting the Notch1 signaling pathway (143). Moreover, BMSCs help to reduce oxidative stress and inflammation *via* down-regulating NF-kB signaling pathway, thereby reducing doxorubicin-induced nephropathy (144). Whether the effect of BMSCs on antioxidant stress in POF is consistent with others or not still needs further research.

### Mitochondrial transfer

Gomzikova et al. have found that MSCs can transfer mitochondria to injured cells through various methods such as tunneling nanotubes, extracellular vesicle and cell fusion, thereby restoring aerobic respiration and mitochondrial function of cells and inhibiting apoptosis (145). Wang L et al. revealed that BMSCs transplantation could resist to mitochondrial dysfunction in age-associated ovarian hypofunction mice through enhancing mitochondrial membrane potential, increasing mitochondrial DNA copies, and improving mitochondrial cristae alignment and vacuolation, as well as regulating expression of mitochondrial dynamics-related proteins (146). Whether it is related to mitochondrial transfer still needs further investigations. Tseng N et al. indicated that when co-cultured with oxidant-damaged neurons, BMSCs could transfer complete mitochondria to injured neurons *via* instantaneous tunneling nanotubes, which may contribute to the preservation and functional recovery of neurons after stroke (147). Human BMSCs can transfer mitochondria to injured human umbilical vein endothelial cells *via* tunneling nanotubes, which helps to promote cell proliferation, reduce cell apoptosis, and enhance their capacity of transmembrane migration and angiogenesis, thereby improving endothelial cells function and hematopoietic system regeneration (148). Furthermore, the mitochondrial transfer induced by BMSC-derived extracellular vesicle helps to enhance phagocytic capacity, decrease secretion of proinflammatory cytokine, and upregulate expression of the M2 phenotype marker CD206 of human macrophages, thereby ameliorating lung injury in the acute respiratory distress syndrome environment (149). In conclusion, mitochondria transfer of BMSCs plays an important role in the treatment of various diseases. Whether BMSCs can recover damaged ovarian function through mitochondrial transfer in POF treatment still needs further investigation.

### Autophagy regulation

Autophagy is a crucial molecular pathway for maintaining cellular and organismal homeostasis, which can remove damaged or excess proteins, organelles, and foreign pathogens in the cells (150). It has been proved that autophagy is involved in the preservation of primordial follicles pools in young mice and the elimination of inferior follicles during follicular development (151). However, Xie QE et al. suggested that excessive autophagy was connected with the pathogenesis of POF (152). Therefore, regulating the activities of autophagy may be an effective method to improve ovarian function of POF (153). Yin et al. indicated that heme oxygenase-1 gene expressed in UMSCs is crucial in restoring the ovarian function of POF mice with UMSCs transplantation by activating JNK/Bcl-2 signaling pathway-regulated autophagy and upregulating the number of CD8+CD28− T cells in the circulation (121). Moreover, Lu X et al. suggested that hUMSCs transplantation could alleviate ovarian function in POF rats *via* inhibiting the theca-interstitial cells apoptosis through reducing autophagy, which achieving in part through regulating ROS levels and inhibiting the AMPK/mTOR signaling pathway (70). From this, we can speculate that maybe BMSCs regulate autophagy to ameliorate ovarian function. Presently, the fact that BMSCs influence autophagy to restore ovarian function in POF has not been reported, but BMSCs do have an effect on autophagy in other diseases, such as ischemia/reperfusion injury (154). Therefore, exploring the mechanism of BMSCs in modulating autophagy may provide a feasible therapeutic strategy for POF.

## Enhancing the effect of BMSCs

Recent years, lots of studies have proved that BMSCs transplantation improves ovarian damage caused by chemotherapy or other factors (13, 49, 50, 53, 56), which provides promising treatment options for POF. However, chronic inflammatory response, hypoxia, oxidative stress and other microenvironmental changes in the damaged tissue area often leads to apoptosis or low homing efficiency of transplanted MSCs (155, 156). Saberi K et al. illustrated that more than 80% of the transplanted cells underwent apoptosis after transplantation to the target organ (157). The low mobility and survival rate of BMSCs transplantation often limit their therapeutic potential. Therefore, enhancing the homing and survival rate of transplanted cells is critical to improve the therapeutic effect of BMSCs (**Table 3**).

**Table 3:** The methods of enhancing MSCs effects.

Methods	Cells type	Intervention way	Main effects	Reference
Overexpression CXCR4	BMSCs	Gene modification	Promoted the homing of BMSCs to injured sites.	Zheng XB et al., 2019 (80), Chen L et al., 2020 (86)
Overexpression SDF-1	BMSCs	Gene modification	Promoted BMSCs migration, proliferation and osteogenic differentiation by activating the Wnt pathway.	Meng Z et al., 2021 (85)
Overexpression CMET	BMSCs	Gene modification	Promoted the homing of BMSCs to injured sites.	Wang K et al., 2017 (94)
Overexpression miR-21	BMSCs	Gene modification	Decreased BMSCs apoptosis. Promoted efficacy against chemotherapy-induced POF.	Fu X et al., 2017 (139)
Co-overexpression CXCR4 and IL-35	BMSCs	Gene modification	Enhanced migration and immunomodulatory activity of BMSCs.	Tan C et al., 2022 (158)
co-overexpression VEGF and Bcl-2	BMSCs	Gene modification	Protected BMSCs by inhibiting apoptosis, suppressing autophagy and enhancing the paracrine effects.	Ni X et al., 2017 (159)
Overexpression PARK7	BMSCs	Gene modification	Increased antioxidative-stress processes and survival of BMSCs *via* activating ERK1/2 signaling pathway. Enhanced resistance to oxidative stress and inhibited stress-induced apoptosis in BMSCs by regulating Nrf2 signaling pathway.	Zhang F et al., 2020 (160), Zhang F et al., 2021 (161)
G-CSF	BMSCs	Coadministration/ pretreatment	The efficacy of the coadministration of BMSCs and G-CSF in restoring ovaries damaged was more effective. Promoted the homing of BMSCs *via* upregulating CXCR4 expression	Sameni HR et al., 2019 (48), Zhao F et al., 2019 (162)
Shikonin	UMSCs	Coadministration/pretreatment	Regulated autophagy *via* AMPK/mTOR pathway and reduced apoptosis of human UMSCs to improve survival in injured site.	Zhu X et al., 2021 (163)
Starvation medium	ADMSCs	pretreatment	Increased the immunomodulation	Vu BT et al., 2021 (164)
Erythropoietin	BMSCs	pretreatment	Increased the homing ability of BMSCs. Protected BMSCs from apoptosis through SIRT1 pathway. Reduced the lung entrapment of BMSCs and increased distribution in target organs. Enhanced therapeutic of BMSCs.	Zhou S et al., 2018 (165), Zhou S et al., 2020 (166)
H2O2	BMSCs	pretreatment	Promoted homing to injured site.	Guo L et al., 2020 (82)
Hypoxia	BMSCs	pretreatment	BMSCs increase VEGF, FGF2, HGF, and IGF-1 expression by nuclear factor-kappa B mechanism.	Lahm T et al., 2008 (167)
LIPUS	ADMSCs BMSCs AMSCs	Treatment/pretreatment	Maintained ADMSCs stem-cell property by activating MAPK/ERK and PI3K/AKT signaling pathways by up-regulating CyclinD1 gene and c-myc gene in ADMSCs cells. Promoted cells proliferation by activating PI3K/AKT and ERK1/2 signaling pathways. Promoted expression and secretion of growth factors. More advantageous for reducing inflammation, improving local microenvironment and inhibiting GCs apoptosis in ovarian tissue of POF.	Huang D et al., 2020 (168), Xie S et al., 2019 (169), Ling L et al., 2017 (170), Ling L et al., 2017 (171)
HSP	BMSCs UMSCs	pretreatment	Alleviated the apoptosis and improve the survival of BMSCs through elevating expression of HSP70 and HSP90 and attenuating autophagy. Enhanced inhibitory effect of UMSCs on inhibiting NLR family pyrin domain containing 3 inflammasome activation in macrophages by upregulating HSP70.	Chen X et al., 2018 (27), Wang Q et al., 2019 (172), Lv H et al., 2021 (173)
L-carnitine	BMSCs	treatment	Suppressed cell apoptosis by elevating ATP. Inhibited adipogenic differentiation.	Fujisawa K et al., 2017 (174)
Apigenin	BMSCs	coadministration	More effectively restored damaged ovaries. May increase the differentiation of transplanted BMSCs.	Talebi A et al., 2020 (175)
PRP	UMSCs	coadministration	Increased the survival rate of UMSCs transplantation. Enhanced the beneficial effects of UMSCs in treating POF.	Wang J et al., 2021 (176)
Collagen scaffolds	ADMSCs	Co-transplantation	Increased the short-term retention of ADMSCs in ovaries and contributed to long-term restoration of ovarian function	Su J et al., 2016 (177)
PLGA/MH sponge or hyaluronic acid gel type of scaffold	ESC-MPCs	Co-transplantation	Effectively prolonged the cell survival rate *in vivo*. Exhibited the best recovered ovarian functions.	Shin EY et al., 2021 (178)
Programmable microencapsulation	BMSCs	Co-transplantation	enhanced BMSCs persistence and immunomodulation.	Mao AS et al., 2019 (179)

AMSCs, amniotic mesenchymal stem cells; ADMSCs, adipose-derived mesenchymal stem cells; BMSCs, bone marrow mesenchymal stem cells; ESC-MPCs, embryonic stem cell-derived mesenchymal progenitor cells; FGF2, fibroblast growth factor2; G-CSF, granulocyte colony-stimulating factor; HGF, hepatocyte growth factor; HSP, heat shock pretreatment; IGF, insulin-like growth factor; LIPUS, low-intensity pulsed ultrasound; PARK7, Parkinson’s disease protein 7; POF, premature ovarian failure; PRP, platelet-rich plasma; UMSCs, umbilical cord mesenchymal stem cells; VEGF, vascular endothelial growth factor.

### Gene-modified BMSCs transplantation

To enhance repairing capabilities of transplanted cells, gene modification is worth to take into consideration before BMSCs transplantation. Previous studies indicated that overexpression of homing related factors, such as SDF1, CXCR4, CMET, can help to promote the homing of BMSCs significantly (85, 86, 94). After dual genetic modification of BMSCs *via* transducing CXCR4 and IL-35, the migration capability and immunoregulation effects of BMSCs are remarkably improved compared to their natural counterparts (158), which implies that dual CXCR4/IL-35 overexpression-BMSCs may be a promising and attractive treatment in autoimmune POF. Fu X et al. revealed that miR-21-overexpression BMSCs showed less apoptosis and more vitality after transplanting and stronger repairing effect in chemotherapy-induced POF compared with the transplantation of BMSCs injection alone (139). Ni X et al. found that co-overexpression of VEGF and Bcl-2 protected BMSCs from a hostile environment through inhibiting apoptosis, suppressing autophagy and enhancing paracrine signaling (159). In addition, Parkinson’s disease protein 7 (PARK7) is an antioxidant protein that enhances cellular resistance to oxidative stress and stress-induced apoptosis (180, 181). PARK7 overexpression enhances antioxidative‐stress capacity in BMSCs *via* activating the ERK1/2 signal pathway, which effectively decreases the level of ROS/malondialdehyde and protects the mitochondrial membrane potential as well as inhibits apoptosis of BMSCs subjected to oxidative stress (160). Moreover, PARK7 can also promote the disintegration of Nrf2/Kelch-like echinacoside associated protein 1 complex, thereby activating Nrf2. And then the activated Nrf2 will enter the nucleus to activate the expression of manganese superoxide dismutase, catalase, glutathione peroxidase, and other antioxidant enzymes. This cascade helps to remove excessive cellular ROS and protect BMSCs from stress-induced apoptosis (161). Therefore, BMSCs overexpressing PARK7 before transplantation may help to reduce BMSCs apoptosis induced by oxidative stress and increase the homing efficiency of BMSCs to the damaged microenvironment, thereby enhancing their repairing effect. From this, gene‐modified BMSCs transplantation may be a promising method to enhance the therapeutic effect of BMSCs.

### Pretreatment and co-transplantation

Studies have reported that when the transplanted cells are trapped in an undesirable environment, such as free radicals, inflammation or hypoxia, they may suffer early death, which may limit the beneficial effects of these cells (157). Thus, it will be beneficial to develop a special pre-treatment system or co-transplantation to enhance the proliferation, homing and survival ability of BMSCs to improve their performance. Zhu X et al. revealed that shikonin pretreatment significantly inhibited apoptosis in hUMSCs under hypoxic-ischemic conditions *in vitro* or vivo studies through regulation of autophagy *via* regulating AMPK/mTOR signal pathway, thereby improving hUMSCs survival surrounding injured tissues (163). In addition, the immunoregulation capability of human ADMSCs can be changed when cultivated under serum starvation to adapt to the different culture conditions *in vitro (* 164), which suggests that serum starvation pretreatment may be beneficial to enhance the therapeutic effect of transplanted BMSCs. Cunningham CJ et al. revealed that hypoxic pretreatment of BMSCs could increase the secretion of VEGF, FGF2, HGF, IGF1 (167). Erythropoietin (EPO) is a glycoprotein hormone with the effect of antioxidant and anti-inflammatory. Vitro studies showed that the proliferation rate and mobility of BMSCs were significantly increased after 48h pretreatment with 500 IU/mL EPO, which may be achieved by regulating BMSCs cytoskeletal rearrangement and upregulating the expression of CXCR4 (165). *In vivo* studies also confirmed that pretreatment with EPO before transplantation significantly increased the homing and therapeutic capacity of BMSCs (165). In addition, EPO is related to the activation of SIRT1 signal in BMSCs, and then regulates the expression of P53 and Bcl-2, which shows an anti-apoptotic effect (166). Colony-stimulating factors, which are hematopoietic growth factors, participate in regulating the proliferation, migration and differentiation of bone marrow cells. BMSCs pretreated with granulocyte colony-stimulating factor help to enhance the homing efficiency of BMSCs to injured tissues by upregulating the CXCR4 expression, which significantly increases repairing effects of BMSCs (162). Sameni HR et al. also confirmed that the recovery of ovarian function in POF rats was more favorable in coadministration of BMSCs with granulocyte colony-stimulating factor compared with the administration of either of them individually (48).

Low intensity pulsed ultrasound (LIPUS) is a pulse emission with low intensity and low thermal effect. Studies have found that LIPUS exposure can activate MAPK/ERK and PI3K/Akt signal pathways by up-regulating CyclinD1 and C-MYC genes, thus promoting the proliferation of MSCs (168, 169). Ling L et al. also indicated that the activation of ERK1/2 and PI3K/Akt signal pathways may be one of the potential mechanisms of LIPUS promoting the proliferation of MSCs (170). Animal studies also found that compared with human ADMSCs transplantation, LIPUS-pretreated human ADMSCs transplantation could not only repair chemotherapy-induced ovarian damage and improve ovarian function in POF rats, but also show greater advantages in alleviating ovarian tissue inflammation, improving local microenvironment and inhibiting chemotherapy-induced GCs apoptosis (171). Heat shock pretreatment (HSP) is an effective way to protect cells before and after transplantation. Recent studies have shown that HSP can inhibit apoptosis and improve the survival of BMSCs in the chemotherapy environment. The mechanism may be related to the elevated expression of HSP90 and HSP70 and the reduction of autophagy (172). In addition, HSP can also enhance the immunomodulatory ability of MSCs (173), which may enhance the therapeutic potential of BMSCs. Studies indicated that melatonin, L-carnitine, apigenin pretreatment could remarkably improve the homing and survival of BMSCs and improve the beneficial effects of MSCs therapy (50, 157, 174, 175, 182). Wang J et al. found that human cord blood platelet-rich plasma could promote proliferation and reduce apoptosis of hUMSCs. Co-transplantation hUMSCs with human cord blood platelet-rich plasma could increase the number of MSCs homing to the ovary of POF rats, which more effectively restored the estrous cycle and repaired damaged follicles of POF rats (176). In addition, Xu H et al. indicated that co-transplantation of BMSCs and endothelial progenitor cells could promote angiogenesis in the site of osteonecrosis of the femoral head (183). From this, we speculate that maybe co-transplantation BMSCs with endothelial progenitor cells can significantly ameliorate ovarian function in POF in the same way, which deserves further exploration.

### BMSCs and biomaterials

Biomaterials take the advantages of promoting cell interactions, excellent stability and biodegradability, good passive and active targeting and show great potential in various applications including regenerative medicine (184). Su et al. reported that ADMSCs transplantation by collagen scaffold increased the retention of MSCs in ovaries and contributed to long time restoration of ovarian function in the POF rat model (177). However, transplanting cells directly into the core of the ovaries may lead to ovarian injury resulting from needle puncture. Shin EY et al. effectively avoid the disadvantage through subcutaneous transplanted MSCs using hyaluronic acid gel scaffold, and at the same time, the method effectively prolongs the survival rate of transplanted cells and significantly recovers ovarian functions in POF rats, too (178). Moreover, Mao AS et al. found that biomaterial encapsulation of BMSCs into programmable microencapsulation using a microfluidic device could partly reduce donor rejection and efficiently increase retention *in vivo* after intravenous injection, which substantially sustained BMSCs survival and enhanced overall immunomodulatory capacity of BMSCs in a model of allogeneic transplantation (179).

## Exosomes

Although numerous studies have shown that BMSCs transplantation can effectively restored the ovarian structure and function of POF, there are still some limitations, such as invasive operation, uncontrollable preparation quality, immunological rejection, post-transplantation infection, secondary injury, descendant safety, chromosomal aberration, potential tumorigenicity (185–187). Chen S et al. demonstrated that the repairing effects of BMSCs and their exosomes were consistent in reducing senescent and apoptotic of GCs after phosphoramide mustard injury (21). From it, it’s worth to explore whether BMSCs-derived exosomes can provide a new strategy and direction for restoring POF ovarian function and at the same time avoid the disadvantages of direct BMSCs transplantation.

Exosome is a vesicle with a diameter of 40-100 nm secreting by cells and contains various proteins, mRNA and microRNAs, which is involved in cell communication and migration, angiogenesis and growth of tumor cells (37, 187). Yang M et al. revealed that BMSC-derived exosome miR-144-5p-mediated PTEN inhibition resulted in increasing PI3K/AKT signal activation, which was conducive to decrease GCs apoptosis and increase ovarian reserve in chemotherapy-induced POF (37). Similarly, the delivery of BMSC-derived exosome miR-644-5p to GCs plays a key role in regulating p53 expression of cells and thereby inhibiting GCs apoptosis and restoring ovarian function in cisplatin-induced POF mice model (188). MiR-126 is an important regulator for the function of endothelial cells and angiogenesis (189). BMSCs-derived miR-126 can enhance the survival and angiogenic function of injured endothelial cells by activating the PI3K/Akt/eNOS pathway and reducing cleaved caspase-3 expression, while increasing the expression of VEGF, epidermal growth factor, platelet derived growth factor, bFGF and other angiogenesis and growth factors (190). In addition, human BMSCs-derived exosomes can induce tubule formation *in vitro* and promote angiogenesis through NF-kB signaling pathway (191). Related studies have shown that exosomes secreted by MSCs from different tissues such as amniotic membrane and umbilical cord also play an important role in POF treatment. hUMSCs-derived exosome helps to rescue POF and reduces ROS accumulation by downregulating the expression of SIRT7 and its downstream target genes *via* delivering exosome miR-320a (192). Similarly, hUMSCs-derived exosome can recover ovarian structure and function, promote GCs proliferation and inhibit ROS accumulation in CTX -induced POF mouse model by down-regulating the expression of SIRT7 and its downstream target genes (PARP1, γH2AX and XRCC6) *via* delivering miR-17-5p (193). Xin Mi et al. also found that the secretome of hUMSCs helped effectively to activate primordial follicle both *in vivo* and vitro. And furthermore, hUMSCs-derived HGF upregulated the expression of KIT ligand in GCs, thereby promoting the activation of PI3K/Akt signaling pathway in dormant oocytes (194). Li Z et al. revealed that hUMSCs-derived exosome significantly ameliorated ovarian function and reproductive ability of POF mice models through promoting GCs proliferation *via* the Hippo signaling pathway (195). Cai et al. also found that hUMSCs-derived miR-21 could inhibit the expression of LATS1, so as to reduce phosphorylated LOXL2 and YAP, and ultimately promote E2 secretion in ovarian GCs (196). Moreover, hUMSCs-derived miR-126-3p promote proliferation while inhibit the apoptosis of GCs through PIK3R2/PI3K/AKT/mTOR pathway (197). Similarly, Yang et al. demonstrated that hUMSCs exosome transplantation could ameliorate ovarian function through promoting angiogenesis by activating PI3K/AKT signaling pathway (198).

In a word, the above studies have proved that exosomes derived from MSCs effectively ameliorate ovarian function in POF by promoting angiogenesis, inhibiting oxidative stress, suppressing cell apoptosis, and exerting many beneficial effects, which may represent a prospective cell-free therapy for developing therapeutic regimen for POF (**Table 4**). But there are still some limitations. Firstly, there is no standardized methods to produce enough exosomes. Moreover, the exosomes transplanted into the body are quickly degraded and lost. Finally, whether exosome therapy alone is equivalent to stem cell treatment is still uncertain (199). All in all, how to avoid the drawbacks of BMSCs transplantation effectively or find a better alternative method still needs further research.

**Table 4:** Therapeutic effect of exosomes derived from MSCs in POF.

Sources	Contents	Function	Reference
BMSCs-EVs (human)	N/A	Reduced the proportions of senescent and apoptotic GCs after phosphoramide mustard injury.	S. Chen et al.2020 (21)
BMSCs-EVs (rat)	miR-144-5p	Inhibited GCs apoptosis. Preservation of ovarian follicles after chemotherapy-induced POF through the PTEN/PI3K/AKT axis.	M. Yang et al., 2020 (37)
BMSCs-EVs (mouse)	miR-644-5p	Inhibited ovarian GCs apoptosis by targeting regulation of p53.	B. Sun et al., 2019 (188)
BMSCs-EVs (mice)	miR-126	Enhanced endothelial cells proliferation, migration, and tube formation *via* activating the PI3K/Akt/eNOS signaling pathway. Inhibited endothelial cells apoptosis by downregulation of cleaved caspase-3. Enhanced the expression of FGF, bFGF and angiogenic factors (PDGF, VEGF) in injured endothelial cells.	Q. Pan et al., 2019 (190)
BMSCs-EVs (human)	Angiogenic paracrine effectors (PDGF, EGF, FGF)	Induced proangiogenic factors expression in endothelial cells. Modulation of angiogenesis *via* nuclear factor-kappa B signaling.	J. D. Anderson et al., 2016 (191)
AMSCs-EVs (human)	miR-320a	Resistance to ovarian senilism. Reduced the ROS levels by regulating SIRT4 by delivering exosomal miR-320a to oocyte, hGCs and ovaries.	C. Ding et al., 2020 (192)
UMSCs-EVs (human)	miRNA-17-5p	Promoted proliferation of CTX-damaged hGCs and ovarian cells. Alleviated ROS accumulation. Improved ovarian function in POF by regulating SIRT7.	C. Ding et al., 2020 (193)
UMSCs-EVs (human)	HGF	Promoted primordial follicle activation by increasing the activity of the PI3K-AKT signaling pathway.	X. Mi et al., 2022 (194)
UMSCs-EVs (human)	N/A	Improved ovarian functions and proliferation by regulating the Hippo pathway. Promoted the proliferation of ovarian GCs.	Z. Li et al., 2021 (195)
UMSCs-EVs (human)	miR-21	Promoted estrogen production in ovarian GCs *via* LATS1-mediated phosphorylation of LOXL2 and YAP.	J. H. Cai et al., 2022 (196)
UMSCs-EVs (human)	miR-126-3p	Promoted angiogenesis and attenuated ovarian GCs apoptosis in POF.	Q. Qu et al., 2022 (197)
UMSCs-EVs (human)	N/A	Restored ovarian function by inducing angiogenesis *via* the PI3K/AKT signaling pathway.	Z. Yang et al., 2019 (198)

AMSCs, amniotic mesenchymal stem cells; BMSCs, bone marrow mesenchymal stem cells; bFGF, basic fibroblast growth factor; EGF, epidermal growth factor; EVs, exosomes; FGF, fibroblast growth factor; GCs, granulosa cells; HGF, hepatocyte growth factor; miR, microRNA; POF, premature ovarian failure; ROS, reactive oxygen species; UMSCs, umbilical cord mesenchymal stem cell.

## Discussion

POF is a common endocrine disorder that causes infertility in women, which affecting about 0.1% and 1% of women under 30 and 40 years old, respectively. POF is irreversible and currently incurable. Currently, HRT is the preferred treatment for POF, but it is unclear whether HRT increases the risk of breast cancer and venous thromboembolism (200). Therefore, it is crucial to find a better treatment for POF patients. Studies reveal that BMSCs have great potential for alleviating POF in laboratory-based investigations and pre-clinical as well as clinical studies in the last decade (64, 201, 202). BMSCs transplantation is a terrific option for POF treatment due to their low immunogenicity, availability and broad sources (35, 37). They can improve POF ovarian function through various mechanisms including paracrine, angiogenesis, anti-fibrosis, anti-inflammatory and immune regulation, anti-oxidative stress, inhibition of apoptosis, mitochondrial transfer and autophagy regulation after homing to the damaged ovary. At the same time, more and more researchers focus on optimizing the efficacy and safety of MSCs through genetic modification, pretreatment or co-transplantation and biomaterials processing. Moreover, exosomes secreted by MSCs may be a prospective cell-free therapy for developing therapeutic option for POF.

BMSCs have great therapeutic potential in many diseases, however, there are still some limitations. First and foremost, the specific markers of MSCs are unclear, so it is difficult to purify MSCs. Next, the mechanism of MSCs homing is still not fully understood, especially the mobilizing mechanism of MSCs, therefore targeted transplantation of BMSCs to injured ovaries remains a challenge (203). Thirdly, the existence of certain biosafety and biological efficacy concerns of MSCs may restrict their clinical applications, including tumor formation, chromosomal aberration, and immunological rejection (37, 186). Moreover, failure of homing to the target damaged tissue precisely and efficiently may lead to some fatal complications such as pulmonary embolism and induced thromboembolism (204–206). More than that, cell senescence is still the bottleneck for clinical applications of BMSCs. Last but not least, there is still no unified standard for BMSCs treatment. For instance, the dosage of stem cell transplantation and the choice of transplantation method, intravenous, orthotopic or intraperitoneal injection? Libing Shi et al. found that intraovarian injection may effectively improve the utilization of MSCs in the ovaries and reduce the adverse effect to other organs, nevertheless, the data also found that intraovarian hUMSCs injections may be toxic to the ovaries and oviduct, which manifested as ovarian lesions and inflammatory cell infiltration in the ovaries (206). In addition, although MSCs have demonstrated obvious therapeutic efficacy in animal models of POF, it is not sufficient to ensure that the majority of patients with POF regain their ovarian reserve in clinical (207).

In view of the problems existing in BMSCs transplantation, it is urgent to seek positive solutions. Firstly, improving standardization of BMSCs including manufacturing protocols, therapeutic targets, dosage, delivery strategy, number, and treatment protocols may partly avoid transplanted relevant risks such as thromboembolism and immunological rejection. Secondly, the tumorigenicity of BMSCs transplantation has long been a concern. Park HS et al. indicated that the number of transplanted cells gradually decreased and almost disappeared after 4 weeks in POF ovaries (53), therefore, the transient residence time and low survival of exogenous BMSCs after transplantation *in vivo* decrease the risk of tumorigenesis (186). Not only that, previous studies also revealed that BMSCs was safe at least six months after transplantation and without significant found of tumorigenicity (146). In addition, Zhang S et al. revealed that ovarian regenerative patch that composed by clinically relevant hydrolysable scaffolds and synthetic MSCs, which encapsulated the secretome of MSCs, restored ovarian function and rescued fertility in POF rats efficiently. The strategy could efficiently avoid the drawback of direct implantation of live cells that increased the risk of developing tumor and immunological rejection, and at the same time, it was able to maintain secretome of MSCs sustainable release to play a more effective therapy (208), which may provide a clinically feasible treatment for POF.

## Conclusion

In summary, BMSCs have undergone a long process of scientific research and exploration from initial discovery to gradual application in the treatment of clinical diseases. Long-term studies found that BMSCs show great potential in ameliorating POF ovarian function through homing, paracrine, angiogenesis, anti-fibrosis, anti-inflammatory and immune regulation, anti-oxidative stress, inhibition of apoptosis, mitochondrial transfer and autophagy regulation. And genetic modification, pretreatment, co-transplantation and biomaterials processing effectively enhance the therapeutic effects of BMSCs. In addition, exosome is a prospective cell-free therapy in POF. Up to now, more and more clinical trials of MSCs in the treatment of POF have been gradually carried out (59, 209) (**Table 5**), but there are still many details which need to be further improved and explored. Formulating systematic standards of BMSCs from culture to application can help to increase the safety of BMSCs-based applications and avoid the side effects. The application of gene modification, biomaterial and exosomes may bring good news to the treatment of POF. It is believed that BMSCs-mediated therapy has broad application prospect for fundamental restoration of ovarian function in POF patients.

**Table 5:** Clinical trials of MSCs treatment for POF.

Identifier	Patients	Study design	Source of MSCs	Delivery route
NCT02696889	Female over the age of 18. Diagnosis of POF. Normal karyotype 46, XX. Presence of at least one ovary. Acceptable uterine anatomy. Normal thyroid function. No other causes of female infertility.	N/A	Autologous bone marrow	intraovarian
NCT02779374	Women with POF: Less than 40 years. amenorrhea of at least 4 months. FSH level above 25 IU/L (repeated twice >4 weeks apart).	N/A	Autologous bone marrow	intravenous
NCT04675970	Diagnosed of POF ESHRE: Women age of 18-40 years. Have experienced 4 months of oligo/amenorrhea. Two serum follicle-stimulating hormone (FSH was >40 mIU/ml levels in the menopausal range, obtained at least a month apart. Lower FSH levels (25 mIU/ml). AMH serum levels (3.0 ng/ml).	N/A	Autologous bone marrow	N/A
NCT03069209	Female. Age: 20-39 years old. FSH>20 IU/L.	Phase 1/2	Autologous bone marrow	intraovarian
NCT02062931	Post-menarche female less than 40 years old. FSH more than or equal to 20 IU/L. Female with normal karyotyping.	Phase 1/2	Autologous bone marrow	intraovarian
NCT02372474	Post-menarche female less than 40 years old. Normal karyotyping female. Primary ovarian failure females FSH more than or equal to 20 IU/L.	Phase 1/2	Autologous bone marrow	intraovarian
NCT03816852	Meet diagnostic criteria of ESHRE. No hormonotherapy and Chinese traditional medicine within 3 months.	Phase 2	Human umbilical cord	intravenous
NCT05308342	Meet the POF diagnostic criteria and have no spontaneous follicular activity. Married, 20 years old ≤ age < 40 years old. The average diameter of each ovary is > 10 mm.	N/A	Human umbilical cord	intraovarian
NCT02644447	Diagnosed with POF. Patients show no response to drug treatment. Women between 20 and 39 years.	Phase 1/2	Human umbilical Cord	intraovarian
NCT01742533	Between age 18- 39 years, Female only. Diagnosed with POF, and currently receiving Hormone Replacement Therapy.	Phase 1/2	Human umbilical Cord	N/A
NCT03877471	Under 40 years of age. Have established regular menstrual cycle, oligomenorrhea/amenorrhea ≥ 4 months. FSH (Follicle-Stimulating Hormone) > 25 IU/mL. Bilateral ovaries are visible by ultrasound. Have fertility requirement, husband has sperms.	Phase 1	Embryonic stem cell	intraovarian
NCT04706312	Women between35 and 45 years. Diagnosed with Diminished Ovarian Reserve by Bologna criteria, (AFC ≤ 7, or serum AMH level < 1.10ng/ml). failed pregnancies in at least two cycles of *In Vitro* Fertilization or Intracytoplasmic Sperm Injection.	Phase 1	human amniotic MSCs	intravenous
NCT01853501	Clinical diagnosis of POF. Patients show no response to drug treatment. Age between 20 to 39.	Phase 4	Autologous adipose derived stem cells	intraovarian

AMH, anti-mullerian hormone; ESHRE, European Society for Human Reproduction and Embryology; FSH, follicle stimulating hormone; MSCs, mesenchymal stem cells; POF, premature ovarian failure.

## Data availability statement

The original contributions presented in the study are included in the article/Supplementary Material. Further inquiries can be directed to the corresponding authors.

## Author contributions

YH performed the literature search and writing. MZ contributed to draft modification. MMZ, KS and YS contributed to suggestions for revision. All authors contributed to the article and approved the submitted version.

## Funding

This work was supported by the National Natural Science Foundation of China, No. 81904008.

## Acknowledgments

Figures were created with BioRender software (https://biorender.com/).

## Conflict of interest

The authors declare that the research was conducted in the absence of any commercial or financial relationships that could be construed as a potential conflict of interest.

## Publisher’s note

All claims expressed in this article are solely those of the authors and do not necessarily represent those of their affiliated organizations, or those of the publisher, the editors and the reviewers. Any product that may be evaluated in this article, or claim that may be made by its manufacturer, is not guaranteed or endorsed by the publisher.
