# Corrigendum: Bone marrow mesenchymal stem cells in premature ovarian failure: Mechanisms and prospects

**DOI:** 10.3389/fimmu.2022.1108216

**Published:** 2022-12-13

**Authors:** Yanjing Huang, Mengdi Zhu, Zhuo Liu, Runan Hu, Fan Li, Yufan Song, Yuli Geng, Wenwen Ma, Kunkun Song, Mingmin Zhang

**Affiliations:** ^1^ Institute of Integrated Traditional Chinese and Western Medicine, Tongji Hospital, Tongji Medical College, Huazhong University of Science and Technology, Wuhan, Hubei, China; ^2^ Department of Integrated Traditional Chinese and Western Medicine, Tongji Hospital, Tongji Medical College, Huazhong University of Science and Technology, Wuhan, Hubei, China

**Keywords:** premature ovarian failure, mesenchymal stem cells, ovary, ovarian function, bone marrow

In the published article, there was an error in the Funding statement. It was incorrectly stated that “This work was supported by the National Natural Science Foundation of China, No. 8190141809” instead of “This work was supported by the National Natural Science Foundation of China, No. 81904008.” The correct Funding statement appears below.

